# Vegetarian diets and cancer risk: pooled analysis of 1.8 million women and men in nine prospective studies on three continents

**DOI:** 10.1038/s41416-025-03327-4

**Published:** 2026-02-27

**Authors:** Yashvee Dunneram, Jia Yi Lee, Cody Z. Watling, Izabella Lawson, Mahboubeh Parsaeian, Gary E. Fraser, Fayth M. Butler, Dorairaj Prabhakaran, Krithiga Shridhar, Dimple Kondal, Viswanathan Mohan, Mohammed K. Ali, K. M. Venkat Narayan, Nikhil Tandon, Tammy Y. N. Tong, Ruth C. Travis, Tina H. T. Chiu, Ming-Nan Lin, Chin-Lon Lin, Hsin-Chou Yang, Yu-Jen Liang, Darren C. Greenwood, Gillian K. Reeves, Keren Papier, Sarah Floud, Rashmi Sinha, Linda M. Liao, Erikka Loftfield, Janet E. Cade, Timothy J. Key, Aurora Perez-Cornago

**Affiliations:** 1 Cancer Epidemiology Unit, Nuffield Department of Population Health, University of Oxford, Oxford, UK; 2 Human Nutrition and Exercise Research Centre, Faculty of Medical Sciences, Newcastle University, Newcastle upon Tyne, UK; 3 MRC Epidemiology Unit, University of Cambridge School of Clinical Medicine, Cambridge, UK; 4 Division of Cancer Epidemiology and Genetics, National Cancer Institute, Bethesda, MD USA; 5 Centre for Nutrition, Healthy Lifestyles and Disease Prevention, School of Public Health, Loma Linda University, Loma Linda, CA USA; 6 Centre for Chronic Disease Control, New Delhi, India; 7 Emory Global Diabetes Research Center, Woodruff Health Sciences Center and Emory University, Atlanta, GA USA; 8 London School of Hygiene and Tropical Medicine, London, UK; 9 Centre for Health Analytics, Research, and Trends, Trivedi School of Bioscience, Ashoka University, Sonipat, Haryana India; 10 Madras Diabetes Research Foundation (ICMR Collaborating Centre of Excellence) and Dr. Mohan’s Diabetes Specialities Centre (IDF Centre of Excellence in Diabetes Care), Chennai, India; 11 Hubert Department of Global Health, Emory University, Atlanta, GA USA; 12 Department of Epidemiology, Rollins School of Public Health, Emory University, Atlanta, GA USA; 13 Department of Family and Preventive Medicine, School of Medicine, Emory University, Atlanta, GA USA; 14 Department of Endocrinology and Metabolism, All India Institute of Medical Sciences, New Delhi, India; 15 Department of Nutritional Science, Fu-Jen Catholic University, New Taipei City, Taiwan; 16 National Center for Geriatrics and Welfare Research, National Health Research Institutes, Yunlin, Taiwan; 17 Department of Family Medicine, Dalin Tzu Chi Hospital, Buddhist Tzu Chi Medical Foundation, Chiayi, Taiwan; 18 Department of Family Medicine, College of Medicine, Tzu Chi University, Hualien, Taiwan; 19 Buddhist Tzu Chi Medical Foundation, Hualien, Taiwan; 20 Institute of Statistical Science, Academia Sinica, Taipei, Taiwan; 21 School of Medicine, University of Leeds, Leeds, UK; 22 Nutritional Epidemiology Group, School of Food Science and Nutrition, University of Leeds, Leeds, UK

**Keywords:** Epidemiology, Cancer epidemiology

## Abstract

**Background:**

Vegetarian diets might influence cancer risk.

**Methods:**

We studied 1,645,555 meat eaters, 57,016 poultry eaters, 42,910 pescatarians, 63,147 vegetarians and 8849 vegans in 9 cohorts (UK, US, Taiwan, India). After a median 16 years follow-up, incident cancers were: 4504 mouth and pharynx, 1308 oesophagus (squamous cell), 2105 oesophagus (adenocarcinoma), 3578 stomach, 30,528 colorectum, 2970 liver, 8030 pancreas, 3077 lung (never smokers), 61,368 breast, 11,220 endometrium, 8076 ovary, 45,946 prostate, 7193 kidney, 6869 bladder, 11,651 non-Hodgkin lymphoma, 4658 multiple myeloma and 7306 leukaemia. Multivariable Cox regression was used to estimate cohort-specific hazard ratios (HRs) and 95% confidence intervals (CIs), and the results were combined using meta-analysis.

**Results:**

Compared to meat eaters, poultry eaters had lower risk of prostate cancer (0.93, 0.88–0.98), pescatarians had lower risks of colorectal (0.85, 0.77–0.93), breast (0.93, 0.88–0.98) and kidney cancer (0.73, 0.58–0.93), vegetarians had lower risks of cancers of the pancreas (0.79, 0.65–0.97), breast (0.91, 0.86–0.97), prostate (0.88, 0.79–0.97), kidney (0.72, 0.57–0.92) and multiple myeloma (0.69, 0.51–0.93) but higher risk of squamous cell carcinoma of the oesophagus (1.93, 1.30–2.87), and vegans had higher risk of colorectal cancer (1.40, 1.12–1.75).

**Conclusions:**

Vegetarian diets might influence risk for several cancers. The generalisability should be considered cautiously.

## Introduction

Vegetarian diets exclude meat and fish, and vegan diets further exclude dairy products and eggs. Appropriately planned vegetarian and vegan diets are considered to be healthful and nutritionally adequate [1]; compared to omnivorous diets, vegetarian and vegan diets are typically lower in some nutrients such as protein, saturated fat and certain micronutrients such as vitamin B12, but higher in others such as dietary fibre, carotenoids and vitamin C [2, 3]. Such nutritional differences might influence cancer risk, and early though unsubstantiated claims were made that cancer is rare in vegetarians [4]; the first empirical data, from a hospital-based case-control study in India published in 1966, showed that, among non-users of tobacco, vegetarians had a higher risk of oral cancer than non-vegetarians which the author tentatively suggested might be due to malnutrition [5]. Around the same time, interest grew in the role of diet in the aetiology of colorectal cancer; international ecological correlations showed that countries with high intakes of meat generally had high incidence rates [6], and the first case-control study, among Seventh-day Adventists in California, suggested that a lacto-ovo-vegetarian diet may reduce the risk of colon cancer [7]. In subsequent prospective studies in the USA and the UK the risks for colorectal cancer and other specific cancer types in vegetarians compared to meat eaters have varied and overall the results appear inconclusive, probably because none of the studies was large enough to provide adequate statistical power to show convincing evidence for small to moderate differences in risks of individual cancer sites [8–16].

To provide novel evidence on whether vegetarian diets are associated with cancer risk, we established the Cancer Risk in Vegetarians Consortium, bringing together data from prospective studies with large numbers and/or large proportions of participants who follow vegetarian diets [17]. The Consortium is the largest study to date on this topic, and comprises nine cohorts on three continents, with diverse diets and large numbers of incident cancers. Our aim was to examine cancer risk in vegetarians and vegans, as well as in people who eat poultry but not red meat (poultry eaters), and in people who eat fish but not meat or poultry (pescatarians), all compared to meat eaters (eat red and/or processed meat). We investigated cancers of the gastrointestinal tract, lung, reproductive system, urinary tract, and blood. We did not investigate skin cancer or cervical cancer because we did not have information on exposure to their major (non-dietary) causal factors (exposure to UV radiation and HPV, respectively).

## Methods

### Study population

The study design and data harmonisation process have been described in detail elsewhere [17]. Briefly, prospective cohort studies were identified through literature searches and the principal investigators were invited to participate if the cohorts met the following criteria: (1) the cohort had targeted recruitment to include a high proportion of vegetarians (typically >25%), or the cohort was very large (≥500,000 participants) and was therefore likely to include up to ~5000 vegetarians (assuming that ~1% of many populations may be vegetarian); (2) the cohort had reliable follow-up data on cancer occurrence. Eleven studies met these initial inclusion criteria and agreed to participate, and individual participant data were transferred to the University of Oxford for harmonisation and analysis, except for the Tzu Chi Health Study where collaborators conducted separate cohort-specific analyses at the Health and Welfare Data Science Center (HWDC) in Taiwan, using methods aligned with the analyses conducted in Oxford, and shared the results (due to data protection regulations in Taiwan). For the Adventist Health Study-2 (AHS-2), the data transferred were for a subset of the whole cohort, representing participants living in US states where the cancer registry gave permission to share data externally. Of the eleven potentially eligible studies identified, data are reported here for nine: AHS-2 [18], the Center for cArdiometabolic Risk Reduction in South Asia-1 (CARRS-1) [19], EPIC-Oxford [20], the Oxford Vegetarian Study [21], the Tzu Chi Health Study [22], the UK Women’s Cohort Study [23], the Million Women Study [24], the National Institutes of Health-AARP Diet and Health Study (NIH-AARP) [25], and the UK Biobank [26]. Results from the Center for cArdiometabolic Risk Reduction in South Asia-2 (CARRS-2) [19, 27], are not reported here because of the small numbers of incident cancers (<10 cases of any of the cancer sites of interest), and the China Kadoorie Biobank [28] results were not included due to the low stability of vegetarian diet groups during the follow-up (<20% of those classified as vegetarian at baseline reported consuming a vegetarian diet at follow-up) [17].

Prior to data harmonisation, participants were excluded from individual studies based on cohort-specific criteria largely related to data which were missing or outside the expected range. After data harmonisation, we further excluded participants aged 90 or over at recruitment, those with a previous malignant neoplasm (other than non-melanoma skin cancer), no follow-up data, unreliable dietary data (more than 80% missing), and those with implausible energy intakes (women <2092 or >14,644 kJ/day, men <3347 or >16,736 kJ/day; data on energy intakes were available for AHS-2, EPIC-Oxford, the UK Women’s Cohort Study, the Million Women Study and NIH-AARP); full details of exclusions have been published [17]. Each study had approval from their local ethics committee, and all participants provided informed consent at the time of recruitment (in the Oxford Vegetarian Study, UK Women’s Cohort Study and NIH-AARP consent was assumed on the basis of returning a completed questionnaire).

### Diet group classification

Food intake, generally over the previous 12 months or “typical diet”, was assessed at baseline using cohort-specific food frequency questionnaires (FFQs); the number of foods on the FFQs ranged from 16 in the UK Biobank to 217 in the UK Women’s Cohort Study (full details have been published [17]). Using information on the consumption of red meat, processed meat (including processed red meat and processed poultry, but not processed fish), poultry, fish, dairy products and eggs, participants were classified into five diet groups: meat eaters (those who consume any red meat and/or processed meat), poultry eaters (do not consume any red or processed meat but do consume poultry), pescatarians (do not consume red meat, processed meat or poultry, but do consume fish), vegetarians (do not consume red meat, processed meat, poultry or fish, but do consume dairy products and/or eggs), and vegans (do not consume any animal products). Poultry intake was not assessed in the Oxford Vegetarian Study, therefore poultry eaters could not be differentiated from meat eaters in this study. Further details on the classification of diet groups in each cohort have been described previously [17].

Information on dietary intake at resurvey, conducted a median of four to 14 years after baseline, was available for a subsample of participants in all the UK cohorts and CARRS-1; 68-89% of people categorised as vegetarian at baseline were still classified as vegetarian at resurvey, and 12% or fewer vegetarians were re-classified as meat eaters [17].

### Cancer ascertainment

Details of cancer ascertainment in each study are shown in Supplementary Table 1. Incident cancer cases were identified through linkage to cancer registries, except for CARRS-1 where a combination of methods was used (linkage through a cancer registry, and/or self-report, and/or verbal autopsy by trained interviewers at follow-up conducted every 2 years as well as for participants who died [29]). Cancer cases were defined using the World Health Organization’s International Classification of Diseases (ICD)-10 codes [30] (or allocated to these where ICD-9 or ICD-O-3 codes were provided): mouth and pharynx cancer (C00–14), oesophageal cancer (C15) and further divided for cohorts with histological codes (EPIC-Oxford, Million Women Study, NIH-AARP, and UK Biobank) into oesophageal squamous cell carcinoma (ICD-O-3 histological codes 8050–8076) and oesophageal adenocarcinoma (ICD-O-3 histological codes 8140, 8141, 8190–8231, 8260–8263, 8310, 8430, 8480–8490, 8560, 8570–8572), gastric cancer (C16), colorectal cancer (C18–20) [further divided into colon (C18), proximal colon (C18.0–18.5), distal colon (C18.6–18.7), and rectum (C19–20)], liver cancer (C22), pancreatic cancer (C25), lung cancer (C34), female breast cancer (C50), endometrial cancer (C54), ovarian cancer (C56), prostate cancer (C61), kidney cancer (C64), bladder cancer (C67), and lymphatic or haematological cancers (C81–96) further divided into non-Hodgkin lymphoma (C82–85), multiple myeloma (C90), and leukaemia (C91–95). In AHS-2 and NIH-AARP, ICD-O-3 codes (rather than ICD-10 codes) were used to identify malignant cancers and histological codes were used to define lymphatic and haematological cancers (9590–9989), non-Hodgkin lymphoma (9591, 9670–9720), multiple myeloma (9731–9734), and leukaemia (9800–9949) [31]. If a participant was not identified with an incident cancer before death but had cancer as an underlying cause of death, then they were considered to have cancer diagnosed on the date of death.

We describe the results for 17 cancer sites: mouth and pharynx, squamous cell carcinoma of the oesophagus, adenocarcinoma of the oesophagus, stomach, colorectum, liver, pancreas, lung, breast, endometrium, ovary, prostate, kidney, bladder, non-Hodgkin lymphoma, multiple myeloma and leukaemia. The main analyses for lung cancer were restricted to never smokers to avoid residual confounding due to smoking [32]. The results for four subsites of colorectal cancer (colon, proximal colon, distal colon, rectum) are shown in the supplementary materials.

### Covariates

Cohort-specific questionnaires were used to collect baseline data on socio-demographics, smoking, alcohol intake, physical activity, medical history and female reproductive factors; full details of data harmonisation are published [17]. Height and weight were self-reported in the AHS-2, EPIC-Oxford, Oxford Vegetarian Study, UK Women’s Cohort Study, Million Women Study and NIH-AARP, and measured in CARRS-1, Tzu Chi Health Study, and UK Biobank [17]. Body mass index (BMI) was calculated as weight in kilograms divided by height in metres squared.

### Statistical analyses

Characteristics including country, years of recruitment, age at recruitment, average years of follow-up, number of incident cancer cases observed, and number of participants following each dietary pattern were described for each cohort, as were baseline characteristics by sex. For each study and cancer site, multivariable Cox proportional hazards regression models with age as the underlying time variable were used to estimate the hazard ratios (HRs) and 95% confidence intervals (CIs) for poultry eaters, pescatarians, vegetarians, and vegans, with meat eaters (eat red and/or processed meat) as the reference group (all diet groups as defined at baseline). Participants contributed follow-up time from the date of recruitment (or date of the first dietary survey in the Million Women Study) until the date of the first cancer diagnosis, date of death, or date of last follow-up, whichever was the earliest. The models were stratified by sex and by region or method of recruitment, as appropriate. Covariates in the multivariable-adjusted models, all coded as categorical variables, were: cigarette smoking (and tobacco chewing in CARRS-1), alcohol intake, regional and sex-specific height categories, BMI, physical activity, history of diabetes, educational status, living with a partner, ethnic group, and for women parity and ever use of hormone replacement therapy. For female-specific cancers, the models were further adjusted for age at menarche, parity and age at first birth combined, menopausal status, and ever use of oral contraceptives. For prostate cancer, we further adjusted for history of prostate-specific antigen (PSA) screening where available. Details of the categories for covariates are in the supplementary methods; for all the covariates, missing or unknown data were categorised separately as unknown, and the percentages of missing or unknown for each covariate in each cohort are shown in Supplementary Table 2.

To obtain pooled risk estimates across all the cohorts, the logs of cohort-specific HRs were each weighted by the inverse of their variance and combined using a weighted average meta-analysis; this approach, sometimes referred to as ‘fixed effects’, uses weighting for each study approximately proportional to the number of events in that study and does not assume that the true relative risk is the same in all the studies [33]. Heterogeneity across cohorts was assessed using the *I*^2^ statistic (where *I*^2^ values of ~25%, 50% and 75% are considered to indicate low, moderate and high heterogeneity, respectively) and *P* for heterogeneity. Cohorts were included in each cancer site meta-analysis (see details below) if there were at least 10 cases observed of that cancer over the follow-up period, across all the diet groups, and we present results for individual diet groups when there were at least 10 cases of cancer in that diet group, across all the cohorts. For lung cancer, the primary analysis was restricted to never smokers. For breast, endometrial, and ovarian cancers, analyses were restricted to women, while for prostate cancer analyses were restricted to men. For breast cancer, we assessed whether the association between diet group and risk varied by menopausal status at the time of diagnosis; for postmenopausal women, follow-up time was considered from the date of recruitment if they were classified as postmenopausal at baseline, or from when they reached the age of 55 (when ~90% of women are postmenopausal) [34].

To examine the possible influence of reverse causality, where undiagnosed cancer might influence diet, we conducted further analyses excluding the first 4 years of follow-up. To examine potential residual confounding by smoking, we repeated all the main analyses in never smokers. Given that BMI can be considered as both a potential confounder, which was accounted for in the main analyses, and a potential mediator in the causal pathway between diet and the risk of cancer, we also performed analyses without adjusting for BMI.

**Fig. 1:**
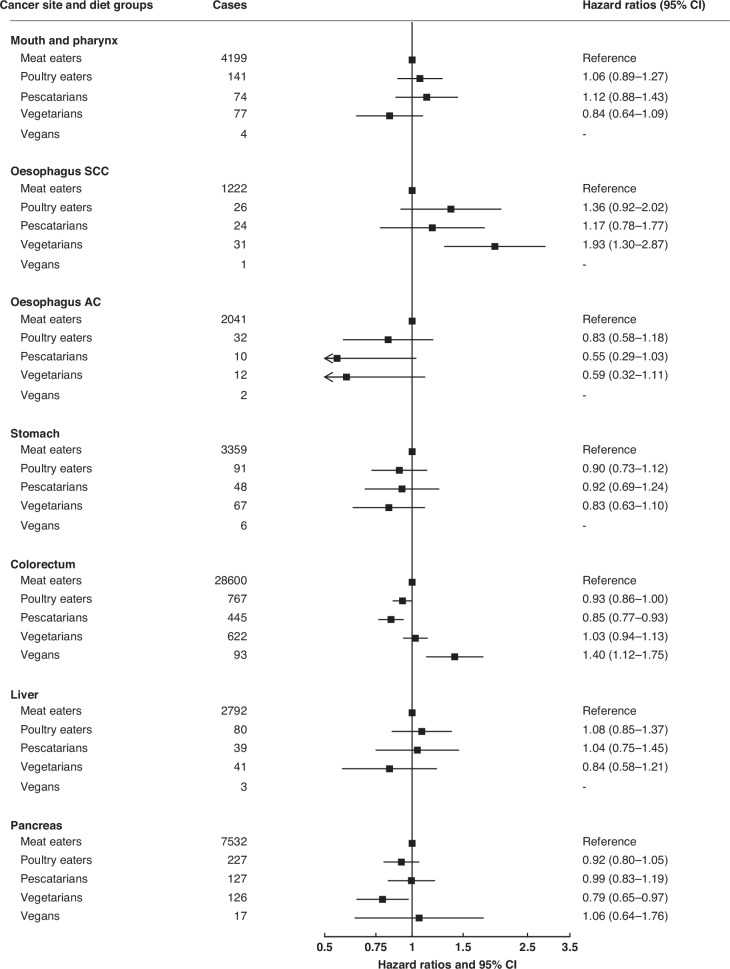
Pooled hazard ratios for cancers of the gastrointestinal tract in poultry eaters, pescatarians, vegetarians and vegans, relative to meat eaters. Results were only reported for diet groups with ≥10 incident cases across all cohorts. Pooled multivariable-adjusted hazard ratios and 95% confidence intervals. The models were stratified by sex and by region or method of recruitment. Covariates in the multivariable-adjusted models were: living with a partner (yes, no), educational status (less than secondary/high school, secondary/high school or equivalent, university degree or equivalent), ethnic group (Asian, Black, Hispanic, White, other), study and sex-specific height categories (women in UK and USA cohorts: <160, 160–164.9, ≥165 cm; women in Asian cohorts: <150, 150–154.9, ≥155 cm; men in UK and USA cohorts: <175, 175–179.9, ≥180 cm; men in Asian cohorts: <163, 163–167.9, ≥168 cm), cigarette smoking history (never, previous, current <10 cigarettes/day, current 10–19 cigarettes/day, current ≥20 cigarettes/day, current unknown number of cigarettes), tobacco chewing (in CARRS-1 only; never, previous, current), physical activity (highly active, moderately active, inactive), alcohol intake (0.0, 0.1–9.9, 10.0–19.9, ≥20.0 g/day), history of diabetes (yes, no), parity (nulliparous, parous), ever used hormone replacement therapy (yes, no), and BMI (<20.0, 20.0–22.4, 22.5-24.9, 25.0–29.9, ≥30.0 kg/m^2^). For all variables, a further category of unknown was included for participants with missing data. ACC adenocarcinoma, SCC squamous cell carcinoma.

**Fig. 2:**
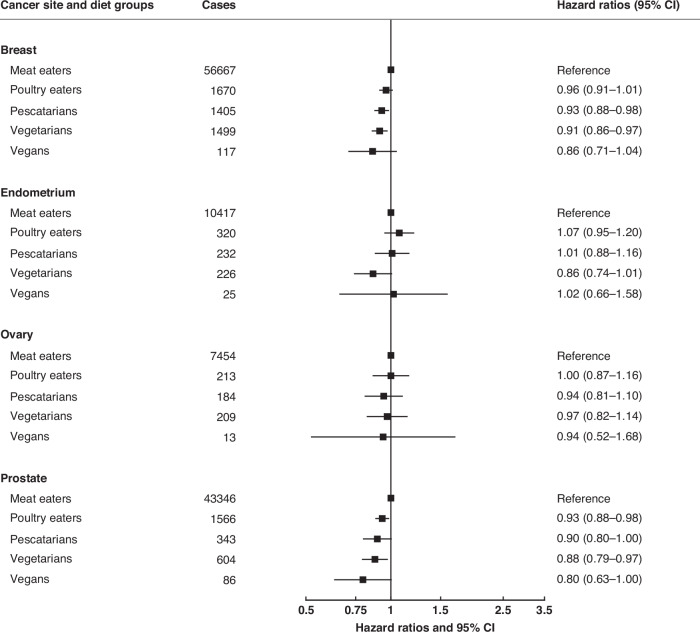
Pooled hazard ratios for cancers of the reproductive system in poultry eaters, pescatarians, vegetarians and vegans, relative to meat eaters. Results were only reported for diet groups with ≥10 incident cases across all cohorts. Pooled multivariable-adjusted hazard ratios and 95% confidence intervals. The models were stratified by region or method of recruitment. Covariates in the multivariable-adjusted models were: living with a partner (yes, no), educational status (less than secondary/high school, secondary/high school or equivalent, university degree or equivalent), ethnic group (Asian, Black, Hispanic, White, other), study and sex-specific height categories (women in UK and USA cohorts: <160, 160–164.9, ≥165 cm; women in Asian cohorts: <150, 150–154.9, ≥155 cm; men in UK and USA cohorts: <175, 175–179.9, ≥180 cm; men in Asian cohorts: <163, 163–167.9, ≥168 cm), cigarette smoking history (never, previous, current <10 cigarettes/day, current 10–19 cigarettes/day, current ≥20 cigarettes/day, current unknown number of cigarettes), tobacco chewing (in CARRS-1 only; never, previous, current), physical activity (highly active, moderately active, inactive), alcohol intake (0.0, 0.1–9.9, 10.0–19.9, ≥20.0 g/day), history of diabetes (yes, no), parity (nulliparous, parous), ever used hormone replacement therapy (yes, no), and BMI (<20.0, 20.0–22.4, 22.5-24.9, 25.0–29.9, ≥30.0 kg/m^2^). For breast, endometrial, and ovarian cancers, the models were further adjusted for age at menarche (≤10 years, 11–12 years, 13–14 years, ≥15 years), parity and age at first birth combined (nulliparous, and parity and age at first birth grouped as: 1–2 and <25 years, 1–2 and 25–29 years, 1–2 and ≥30 years, 1–2 and unknown, ≥3 and <25 years, ≥3 and 25–29 years, ≥3 and ≥30 years, ≥3 and unknown), menopausal status (pre-menopausal, post-menopausal), and ever used oral contraceptives (yes, no). For prostate cancer, the models were further adjusted for history of prostate antigen screening (yes, no). For all variables, a further category of unknown was included.

**Fig. 3:**
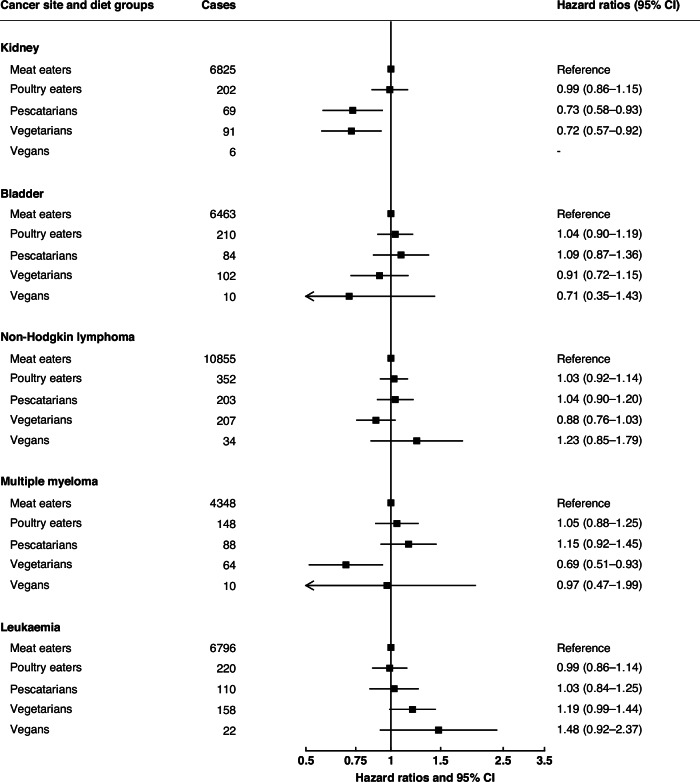
Pooled hazard ratios for cancers of the urinary tract and blood in poultry eaters, pescatarians, vegetarians and vegans, relative to meat eaters. Results were only reported for diet groups with ≥10 incident cases across all cohorts. Pooled multivariable-adjusted hazard ratios and 95% confidence intervals. The models were stratified by sex and by region or method of recruitment. Covariates in the multivariable-adjusted models were: living with a partner (yes, no), educational status (less than secondary/high school, secondary/high school or equivalent, university degree or equivalent), ethnic group (Asian, Black, Hispanic, White, other), study and sex-specific height categories (women in UK and USA cohorts: <160, 160–164.9, ≥165 cm; women in Asian cohorts: <150, 150–154.9, ≥155 cm; men in UK and USA cohorts: <175, 175–179.9, ≥180 cm; men in Asian cohorts: <163, 163–167.9, ≥168 cm), cigarette smoking history (never, previous, current <10 cigarettes/day, current 10–19 cigarettes/day, current ≥20 cigarettes/day, current unknown number of cigarettes), tobacco chewing (in CARRS-1 only; never, previous, current), physical activity (highly active, moderately active, inactive), alcohol intake (0.0, 0.1–9.9, 10.0–19.9, ≥20.0 g/day), history of diabetes (yes, no), parity (nulliparous, parous), ever used hormone replacement therapy (yes, no), and BMI (<20.0, 20.0–22.4, 22.5-24.9, 25.0–29.9, ≥30.0 kg/m^2^). For all variables, a further category of unknown was included.

We describe all the HRs which were nominally statistically significant at two-sided *P* < 0.05, and also indicate HRs which were statistically significant after allowing for multiple testing using the false discovery rate (FDR, among the 16 cancer sites shown in the main Figs. 1 to 3) as defined by Benjamini and Hochberg with a threshold of 0.05 [35]. All statistical analyses were conducted using Stata release 18.1 (StataCorp, College Station, TX, USA). Forest plots were generated using R version 4.1.2 and the package “Jasper makes plots” version 2-266 [36].

## Results

Data were harmonised for 1,817,477 participants in nine prospective studies in four countries, comprising 1,645,555 (90.5%) meat eaters (eat red and/or processed meat), 57,016 (3.1%) poultry eaters, 42,910 (2.4%) pescatarians, 63,147 (3.5%) vegetarians, and 8849 (0.5%) vegans (Table 1). The largest numbers of both vegetarians and vegans were in AHS-2 and EPIC-Oxford, with these two studies contributing 55% of vegetarians and 82% of vegans. The period of recruitment ranged from 1980 to 2010, and age at recruitment ranged from 15 years old and upwards. Mean follow-up across studies ranged from 6 years in CARRS-1 to 27 years in the Oxford Vegetarian Study, and 220,387 incident cancers were identified for the sites of interest: 4504 mouth and pharynx, 1308 oesophagus (squamous), 2105 oesophagus (adenocarcinoma), 3578 stomach, 30,528 colorectum, 2970 liver, 8030 pancreas, 3077 lung (in never smokers), 61,368 breast, 11,220 endometrium, 8076 ovary, 45,946 prostate, 7193 kidney, 6869 bladder, 11,651 non-Hodgkin lymphoma, 4658 multiple myeloma and 7306 leukaemia. Baseline BMI and other characteristics are shown in Table 2; mean BMI in women ranged from 21.8 kg/m^2^ in the Oxford Vegetarian Study to 27.1 kg/m^2^ in AHS-2 and UK Biobank, while mean BMI in men ranged from 22.7 kg/m^2^ in the Oxford Vegetarian Study to 27.8 kg/m^2^ in UK Biobank.

**Table 1:** Details of the cohorts included in the Cancer Risk in Vegetarians Consortium.

Cohort	Country	Recruitment	Age at recruitment (years)	Meat eaters	Poultry eaters	Pescatarians	Vegetarians	Vegans	Years to cancer incidence (median (IQR)	Total incident cancers^b^
*Large proportion vegetarian*										
Adventist Health Study-2	USA	2002–2007	≥30	23,245	10,494	6202	19,389	5225	8 (7–10)	3409
CARRS-1	India	2010–2011	≥20	7214	1958	180	2414	452	6 (5–6)	37
EPIC-Oxford	UK	1993–1999	≥20	26,498	1703	8128	15,433	1990	22 (21–23)	5442
Oxford Vegetarian Study	UK	1980–1984	≥15	5180	-^a^	998	4016	333	27 (14–37)	986
Tzu Chi Health Study	Taiwan	2007–2009	≥18	3519	120	233	1604	44	11 (10–12)	223
UK Women’s Cohort Study	UK	1995–1998	35–69	21,180	929	3804	4075	160	20 (19–21)	2772
*Very large cohorts*										
Million Women Study	UK	1996–2001	50–64	618,450	3414	10,947	6136	79	16 (14–18)	82,003
NIH-AARP	USA	1995–1996	50–69	491,098	32,960	1690	1825	118	16 (10–16)	86,632
UK Biobank	UK	2006–2010	40–69	449,171	5438	10,728	8255	448	12 (11–12)	38,883

Diet group definitions: meat eaters (those who consume any red and/or processed meat), poultry eaters (do not consume any red or processed meat but do consume poultry), pescatarians (do not consume red meat, processed meat or poultry, but do consume fish), vegetarians (do not consume red meat, processed meat, poultry or fish, but do consume dairy products and/or eggs), and vegans (do not consume any animal products).

*CARRS* Center for cArdiometabolic Risk Reduction in South Asia, *EPIC* European Prospective Investigation into Cancer and Nutrition, *NIH-AARP* National Institutes of Health-AARP Diet and Health Study.

^a^The dietary questionnaire did not include a question on poultry, therefore it was not possible to separate poultry eaters from meat eaters.

^b^Total incident cancers for the 17 sites examined in this consortium.

**Table 2:** Baseline characteristics of participants by sex and cohort.

Cohort	*N*	Age at baseline (years), mean (SD)	Height (cm), mean (SD)	BMI (kg/m^2^), mean (SD)	Current smokers, *N* (%)	Alcohol intake (g/day), mean (SD)	University degree or equivalent, *N* (%)	Highly active^a^, *N* (%)
**Women**								
*Large proportion vegetarian*								
Adventist Health Study-2	42,194	57.2 (13.8)	163.3 (7.4)	27.1 (6.4)	410 (0.97)	0.3 (1.6)	20,431 (48.4)	17,753 (42.1)
CARRS-1	6372	41.8 (12.7)	151.7 (5.9)	26.6 (5.3)	58 (0.91)	-	945 (14.8)	1494 (23.4)
EPIC-Oxford	41,494	43.9 (13.8)	164.1 (6.8)	23.6 (4.0)	4439 (10.7)	7.8 (9.8)	16,406 (39.7)	4770 (11.5)
Oxford Vegetarian Study	6480	39.4 (15.6)	163.9 (6.7)	21.8 (2.8)	1049 (16.2)	6.9 (8.4)	434 (6.7)	1831 (28.3)
Tzu Chi Health Study	3329	53.3 (9.8)	156.1 (5.4)	23.3 (3.3)	15 (0.45)	0.2 (4.1)	670 (20.1)	999 (30.0)
UK Women’s Cohort Study	30,148	51.8 (9.3)	163.7 (6.8)	24.4 (4.2)	3,224 (10.7)	8.7 (10.2)	7372 (24.5)	1032 (3.4)
*Very large cohorts*								
Million Women Study	639,043	59.8 (4.9)	162.4 (6.5)	26.2 (4.6)	71,277 (11.2)	5.9 (7.6)	106,496 (16.7)	60,701 (9.5)
NIH-AARP	215,905	61.5 (5.4)	163.3 (6.9)	26.8 (6.0)	30,343 (14.1)	5.7 (14.5)	118,637 (54.9)	34,746 (16.1)
UK Biobank	255,454	56.1 (8.0)	162.5 (6.3)	27.1 (5.2)	22,888 (9.0)	8.7 (11.1)	144,629 (56.6)	43,023 (16.8)
Men								
*Large proportion vegetarian*								
Adventist Health Study-2	22,363	58.0 (13.6)	177.7 (7.9)	26.6 (4.8)	258 (1.2)	0.5 (2.6)	12,713 (56.9)	10,605 (47.4)
CARRS-1	5846	43.6 (13.5)	164.9 (6.9)	24.3 (4.5)	1628 (27.8)	-	1252 (21.4)	1097 (18.8)
EPIC-Oxford	12,444	47.2 (14.4)	177.8 (7.0)	24.2 (3.4)	1648 (13.3)	15.4 (18.0)	6409 (51.6)	2201 (17.7)
Oxford Vegetarian Study	4047	39.7 (15.4)	177.6 (6.7)	22.7 (2.6)	1006 (24.9)	12.7 (12.0)	640 (15.8)	1417 (35.0)
Tzu Chi Health Study	2191	53.6 (10.6)	167.5 (6.0)	24.3 (3.1)	216 (9.8)	2.69 (14.1)	792 (36.0)	834 (37.9)
*Very large cohorts*								
NIH-AARP	311,786	61.8 (5.3)	178.3 (7.5)	27.2 (4.3)	31,497 (10.1)	14.7 (28.7)	207,254 (66.5)	66,132 (21.2)
UK Biobank	218,586	56.6 (8.2)	175.6 (6.8)	27.8 (4.2)	27,580 (12.6)	21.6 (23.5)	136,918 (62.6)	45,905 (21.0)

*BMI* body mass index, *CARRS* Centre for cArdiometabolic Risk Reduction in South Asia, *EPIC* European Prospective Investigation into Cancer and Nutrition, *NIH-AARP* National Institutes of Health-AARP Diet and Health Study.

^a^Highest sex-specific tertile of metabolic equivalents.

“-” indicates that no information was available for this variable in the specified cohort.

The pooled HRs for poultry eaters, pescatarians, vegetarians and vegans compared to meat eaters for 16 cancer sites are shown in Figs. 1 to 3 (excluding lung cancer because this analysis was restricted to never smokers); all the HRs for each individual cancer site by cohorts are shown in Supplementary Figs. 1 to 17.

### Gastrointestinal tract cancers

For colorectal cancer, compared to meat eaters, the HRs were 0.93 (95% confidence interval 0.86 to 1.00) in poultry eaters, 0.85 (0.77 to 0.93, FDR significant) in pescatarians, 1.03 (0.94 to 1.13) in vegetarians, and 1.40 (1.12 to 1.75, FDR significant) in vegans (Fig. 1). In subsite analyses, pescatarians had a lower risk of colon cancer (0.80 (0.71 to 0.90)) and vegans had a higher risk of rectal cancer (1.78 (1.23 to 2.57)) (Supplementary Table 3).

Vegetarians had a higher risk of squamous cell carcinoma of the oesophagus (1.93 (1.30 to 2.87, FDR significant)), but a lower risk of pancreatic cancer (0.79 (0.65 to 0.97)) compared to meat eaters (Fig. 1). Risks for cancers of the mouth and pharynx, adenocarcinoma of the oesophagus, stomach and liver did not vary between meat eaters and the other diet groups (Fig. 1).

### Lung cancer

In our primary analysis, which for lung cancer was restricted to never smokers, the risk of lung cancer did not differ between meat eaters and the other diet groups (Supplementary Fig. 8); in supplementary analyses which included current and ex-smokers (with adjustment for detailed smoking categories), there were lower risks in poultry eaters (0.83 (0.77 to 0.89)) and pescatarians (0.82 (0.72 to 0.92)) than in meat eaters (Supplementary Table 3).

### Cancers of the reproductive system

Compared to meat eaters, risk of breast cancer was lower in pescatarians (0.93 (0.88 to 0.98)) and in vegetarians (0.91 (0.86 to 0.97, FDR significant)) (Fig. 2). These associations were significant in postmenopausal women (pescatarians: 0.91, 0.86 to 0.97; vegetarians: 0.89, 0.83 to 0.95), but not in premenopausal women (Supplementary Table 4).

The risk of prostate cancer was lower in poultry eaters (0.93 (0.88 to 0.98)) and vegetarians (0.88 (0.79 to 0.97)) than in meat eaters. Risks for cancers of the endometrium and ovary did not vary between meat eaters and the other diet groups.

### Cancers of the urinary tract and blood

The risk of kidney cancer was lower in pescatarians (0.73 (0.58 to 0.93)) and in vegetarians (0.72 (0.57 to 0.92)) than in meat eaters. Risk for cancer of the bladder did not vary between meat eaters and the other diet groups (Fig. 3).

Compared to meat eaters, the risk of multiple myeloma was lower in vegetarians (0.69 (0.51 to 0.93)). Risks for non-Hodgkin lymphoma and leukaemia did not vary between meat eaters and the other diet groups.

### Sensitivity analyses; consistency by follow-up time, and results in never smokers

The results for the 11 nominally significant associations identified are shown in Table 3: overall, after excluding the first 4 years of follow-up, and in never smokers. The most consistent findings were the higher risk of squamous cell carcinoma of the oesophagus and lower risk of kidney cancer in vegetarians, which were not attenuated and remained statistically significant in both these sensitivity analyses; the other nine nominally significant associations were not statistically significant in one or both of the sensitivity analyses, although for some of these the HRs changed little.

**Table 3:** Pooled hazard ratios for 11 nominally significant associations, compared to meat eaters^a^: overall, excluding the first four years of follow up, and in never smokers.

Cancer site	Diet group	Main analysis			Excluding first 4 years follow-up		Restricted to never smokers	
		Cases	HR	*I*^2^ heterogeneity between cohorts (*I*^2^, *P*-value)	Cases	HR	Cases	HR
Oesophagus SCC	Vegetarian	31	1.93 (1.30 to 2.87)	0%, *P* = 0.90	25	2.09 (1.34 to 3.26)	22	2.93 (1.82 to 4.73)
Colorectal	Pescatarian	445	0.85 (0.77 to 0.93)	17.4%, *P* = 0.29	357	0.87 (0.78 to 0.96)	247	0.89 (0.78 to 1.01)
Colorectal	Vegan	93	1.40 (1.12 to 1.75)	13.2%, *P* = 0.33	60	1.25 (0.95 to 1.64)	71	1.67 (1.28 to 2.18)
Pancreatic	Vegetarian	126	0.79 (0.65 to 0.97)	0%, *P* = 0.54	95	0.77 (0.61 to 0.97)	79	0.84 (0.64 to 1.10)
Breast	Pescatarian	1405	0.93 (0.88 to 0.98)	0%, *P* = 0.55	1094	0.98 (0.92 to 1.04)	779	0.94 (0.87 to 1.01)
Breast	Vegetarian	1499	0.91 (0.86 to 0.97)	0%, *P* = 0.69	1154	0.96 (0.89 to 1.02)	970	0.95 (0.88 to 1.02)
Prostate	Poultry	1566	0.93 (0.88 to 0.98)	68%, *P* = 0.03	1087	0.91 (0.86 to 0.97)	723	0.98 (0.91 to 1.06)
Prostate	Vegetarian	604	0.88 (0.79 to 0.97)	63%, *P* = 0.03	427	0.93 (0.82 to 1.04)	440	0.99 (0.87 to 1.13)
Kidney	Pescatarian	69	0.73 (0.58 to 0.93)	7%, *P* = 0.37	53	0.70 (0.53 to 0.92)	34	0.72 (0.51 to 1.02)
Kidney	Vegetarian	91	0.72 (0.57 to 0.92)	0%, *P* = 0.97	66	0.70 (0.54 to 0.93)	50	0.64 (0.46 to 0.88)
Multiple myeloma	Vegetarian	64	0.69 (0.51 to 0.93)	18.6%, *P* = 0.29	48	0.75 (0.54 to 1.05)	46	0.76 (0.53 to 1.10)

^a^Eat red and/or processed meat.

Pooled multivariable-adjusted hazard ratios (HRs) and 95% confidence intervals. The models were stratified by sex (where appropriate), and by region or method of recruitment. Covariates in the multivariable-adjusted models were: living with a partner (yes, no), educational status (less than secondary/high school, secondary/high school or equivalent, university degree or equivalent), ethnic group (Asian, Black, Hispanic, White, other), study and sex–specific height categories (women in UK and USA cohorts: <160, 160–164.9, ⩾165 cm; women in Asian cohorts: <150, 150-154.9,⩾155 cm; men in UK and USA cohorts: <175, 175–179.9, ⩾180 cm; men in Asian cohorts: <163, 163-167.9, ⩾168 cm), cigarette smoking history (never, previous, current <10 cigarettes/day, current 10–19 cigarettes/day, current ⩾20 cigarettes/day, current unknown number of cigarettes), tobacco chewing (in CARRS-1 only; never, previous, current), physical activity (highly active, moderately active, inactive), alcohol intake (0.0, 0.1–9.9, 10.0–19.9, ⩾20.0 g/day), history of diabetes (yes, no), parity (nulliparous, parous), ever used hormone replacement therapy (yes, no), and BMI (<20.0, 20.0–22.4, 22.5-24.9, 25.0–29.9, ⩾ 30.0 kg/m2). For breast, endometrial, and ovarian cancers, the models were further adjusted for age at menarche (⩾10 years, 11–12 years, 13–14 years, ⩾15 years), parity and age at first birth combined (nulliparous, and parity and age at first birth grouped as: 1–2 and <25 years, 1-2 and 25-29 years, 1-2 and ⩾30 years, 1-2 and unknown, ⩾3 and <25 years, ⩾3 and 25-29 years, ⩾3 and ⩾30 years, ⩾3 and unknown), menopausal status (pre-menopausal, post-menopausal), and ever used oral contraceptives (yes, no). For prostate cancer, the models were further adjusted for history of prostate antigen screening (yes, no). For all variables, a further category of unknown was included. SCC=squamous cell carcinoma.

### Heterogeneity between cohorts

There was no significant heterogeneity between cohorts for the 11 nominally significant associations with the exception of the lower risks for prostate cancer among poultry eaters and vegetarians (Table 3 and Supplementary Figs. 2, 5, 7, 9, 12, 13, 16, and Supplementary Table 3).

### Impact of adjustment for body mass index (BMI)

The main analyses described above all included adjustment for BMI. To demonstrate the impact of the adjustment for BMI in these models, Supplementary Table 3 also shows the results from multivariable models not including BMI; comparisons showed that, among the 11 nominally significant associations described above, the adjustment for BMI generally modestly attenuated the HRs towards the null (with the exceptions of multiple myeloma in vegetarians, where the HR remained unchanged, and colorectal cancer in vegans, where adjustment for BMI increased the HR from 1.32 to 1.40).

## Discussion

### Principal findings

We harmonised individual participant data from all the identified studies worldwide with information on cancer incidence in substantial numbers of people following vegetarian diets. We examined the risks of 17 types of cancer, comparing poultry eaters, pescatarians, vegetarians, and vegans to meat eaters; poultry eaters had a lower risk of prostate cancer, pescatarians had lower risks of colorectal, breast and kidney cancer, vegetarians had lower risks for cancers of the pancreas, breast, prostate, kidney and multiple myeloma, but higher risk of squamous cell carcinoma of the oesophagus, and vegans had a higher risk of colorectal cancer. In sensitivity analyses, the most consistent findings were that vegetarians had a higher risk of squamous cell carcinoma of the oesophagus and a lower risk of kidney cancer. There was no strong evidence of marked heterogeneity between studies despite the wide geographic spread of the populations.

### Cancers of the gastrointestinal tract

The International Agency for Research on Cancer and the World Cancer Research Fund (WCRF)/American Institute for Cancer Research (AICR) have concluded that the risk for colorectal cancer rises with higher consumption of processed meat, and probably also unprocessed red meat [37, 38]. We observed that, compared to meat eaters, the risk was 15% lower in pescatarians, not different in vegetarians, and 40% higher in vegans. The absence of a lower risk in vegetarians appears inconsistent with an adverse impact of processed and red meat, but it should be noted that processed meat intakes in the meat eating groups in the study populations were moderately low; mean intakes ranged from 2 g/d in the Tzu Chi Health Study to 20 g/d in EPIC-Oxford, with a median across cohorts of ~16 g/d [17], which can be compared with general population data for the UK where mean intakes in 2008–2009 were 34 g/d [39].

The higher risk of colorectal cancer observed in vegans is based on only 93 incident cases among vegans in seven studies in the UK and US, with <10 cases in vegans in five of these studies, and therefore should be interpreted with caution; furthermore, the increased risk was attenuated and no longer statistically significant after excluding the first 4 years of follow-up, although it did remain statistically significant in the analysis restricted to never smokers. This observed increase in risk is not compatible with the predicted reduction in risk due to the absence of meat intake. In all cohorts, vegans had the lowest intakes of alcohol which is a cause of colorectal cancer [40], and the highest intakes of wholegrains and dietary fibre which have been associated with a lower risk [17, 38], suggesting that other aspects of vegan diets in these populations may contribute to the higher risk observed. Vegans have zero intakes of dairy products, and in all cohorts with nutrient intake data vegans had the lowest reported intakes of calcium, mean intakes ranging from 328 mg/d in the Million Women Study to 686 mg/d in the UK Women’s Cohort Study, and a median across the cohorts of 590 mg/d [17], which is low compared to the UK reference nutrient intake for adults of 700 mg/day [41]. The WCRF/AICR concluded that dairy products, and calcium supplements, probably protect against colorectal cancer [38], and a recent diet-wide analysis of colorectal cancer in the Million Women Study showed that the strongest association was with calcium [42], so the higher risk for colorectal cancer in vegans might be due to their low average intake of calcium; low intakes of other nutrients such as long-chain n-3 fatty acids might also be involved [43].

For other cancers of the gastrointestinal tract, vegetarians had a higher risk of squamous cell carcinoma of the oesophagus and a lower risk of pancreatic cancer. Some areas of the world such as northeastern Iran, and Linxian and Cixian in China, have extremely high rates of oesophageal cancer, largely squamous cell carcinoma, which might be linked to various non-dietary factors and/or to restricted diets with low intakes of animal protein, total protein or various micronutrients [44], and recent clinical evidence supports the importance of riboflavin and zinc [45, 46], both of which are abundant in animal foods. Although our findings for squamous cell carcinoma of the oesophagus are based on only 31 cases in vegetarians in three studies in the UK, the risk was of substantial magnitude (1.93) and consistent in our sensitivity analyses.

We observed a lower risk for pancreatic cancer in vegetarians, but this association was attenuated to the null in the analyses restricted to never smokers and should be interpreted with caution; the potential role of diet in relation to pancreatic cancer risk remains unclear [38].

### Lung cancer

The main analyses for lung cancer were restricted to never smokers to avoid residual confounding by smoking; we observed no significant differences in risk between meat eaters and the other diet groups, concordant with recent meta-analyses finding no strong evidence that dietary factors are associated with risk for lung cancer [38]. In the additional analyses of lung cancer in all participants there were small but statistically significant reductions in risk among poultry eaters and pescatarians, probably due to some residual confounding despite the detailed adjustment for smoking history.

### Cancers of the reproductive system

The risk of breast cancer was lower in pescatarians (by 7%) and in vegetarians (by 8%) compared to meat eaters; these differences in risk were confined to postmenopausal women, and were larger before adjusting for BMI, suggesting they may be at least partly due to differences in adiposity. The absence of compelling evidence for differences in breast cancer risk between diet groups after accounting for BMI is consistent with the generally null findings for diet (excluding alcohol) [38, 47]. There was no evidence that risks for endometrial or ovarian cancer varied between meat eaters and the other diet groups. For prostate cancer, risk was 7% lower in poultry eaters and 12% lower in vegetarians compared to meat eaters. Although these associations were attenuated to the null in the analyses restricted to never smokers and should therefore be interpreted cautiously, they appear compatible with the broad hypothesis that lower consumption of animal protein might lead to a reduction in risk of prostate cancer through lower circulating levels of insulin-like growth factor-I [48].

### Cancers of the urinary tract

The risk of kidney cancer was lower in pescatarians (by 27%) and in vegetarians (by 28%) compared to meat eaters. Previous research on meat and kidney cancer risk has been inconclusive [38, 49], but high intakes of animal protein might have adverse impacts on kidney health [50], and circulating concentrations of a biomarker of kidney cancer risk (kidney injury molecule-1 or KIM-1, also called HAVRC1) have been reported to be markedly lower in vegetarians and pescatarians than in meat eaters [51]. Risk of bladder cancer did not vary between meat eaters and the other diet groups, consistent with other null findings on diet [38].

### Cancers of the blood

The risk for multiple myeloma was 30% lower in vegetarians compared to meat eaters. There is a paucity of previous research on diet for this cancer [52]; the only established diet-related risk factor is obesity [53]. Risks for non-Hodgkin lymphoma and leukaemia did not vary between meat eaters and the other diet groups.

### Mechanisms which may link vegetarian diets with cancer risk

In studies from Western Europe and North America, vegetarians typically have several favourable diet-related characteristics, including relatively low intakes of saturated fat and relatively high intakes of dietary fibre, together with low BMI and low low-density lipoprotein cholesterol compared to meat eaters [17, 54]. The lower BMI of vegetarians, observed in all the cohorts except for CARRS-1, would be expected to cause a modestly lower risk for several cancers [53]; all the main results have been adjusted for BMI, thus evaluating the hypothesis that vegetarian diets affect cancer risk independently of differences in BMI.

As well as these favourable characteristics, vegetarians and vegans typically have lower intakes of several nutrients and therefore might be at higher risk of deficiency. In all five cohorts for which we centralised data on nutrient intakes (AHS-2, EPIC-Oxford, UK Women’s Cohort Study, Million Women Study, NIH-AARP), dietary intakes (i.e. excluding any supplements) of protein, vitamin B12, and vitamin D were lower in vegetarians than in meat eaters, and even lower in vegans who also had lower dietary intakes of calcium. Further analyses in EPIC-Oxford have also shown a higher prevalence of inadequate dietary intakes (not taking into account supplements) in vegetarians of bioavailability-adjusted iron, and selenium, and in vegans of vitamin A, riboflavin, zinc and iodine [2], and vegetarians and vegans have low to zero intakes of long-chain n-3 fatty acids [55]. As discussed above, the higher risks we observed for squamous cell carcinoma of the oesophagus in vegetarians, and for colorectal cancer in vegans, might be due to a higher prevalence of inadequate intakes of some nutrients in these groups within the populations studied. Further research could clarify the role of diet in risk of these cancers, and potentially enable mitigation of the excess risks by better food choices, together with food fortification and/or dietary supplements.

### Strengths and limitations

The strengths of the consortium are that it includes all identified prospective studies worldwide with large proportions and/or large numbers of vegetarians, together with long term follow-up for incident cancers. For poultry eaters, pescatarians and vegetarians we had adequate statistical power to detect moderate differences in risks for common cancers, and to detect for the first time substantial differences in risks for the less common cancers; for vegans, however, numbers of cases and therefore statistical power were low. Data harmonisation enabled us to use standardised exposure definitions and regression models for each cancer, and to adjust for major non-dietary risk factors using the same categorisations.

The limitations of this study also need to be considered. We identified and analysed cohorts with large proportions of vegetarians, as well as several very large cohorts (>0.5 million people); a few other moderately large cohorts have published findings on vegetarian diets and cancer risk but with relatively small numbers, for example, 635 vegetarians in the Netherlands Cohort Study (compared to ~63,000 in the current analysis) [56]. Within the UK and the USA there might be some overlap of participants between the cohorts, but we do not have information on this and any overlap is likely to be minor. Some of the nominally significant associations observed might be due to chance because of the number of tests performed, but we interpreted all the findings cautiously.

Vegetarian diets are defined by the foods that are not eaten rather than by the foods that are eaten, so while vegetarian diets are often high in foods thought to be healthy (e.g. fruit and vegetables), they can be high in less healthy foods such as highly refined carbohydrates, and we do not have data on food processing or cooking methods; correspondingly, the meat and poultry eaters, and the pescatarians, in the contributing cohorts may not eat large amounts of the foods which define their diet group [17]. Furthermore, there may be some misclassification of diet group at baseline, and we did not assess duration of diet adherence before participants joined the cohorts, although where these data are available in individual cohorts the majority of vegetarians had followed their diet for at least several years and analyses of nutritional biomarkers, for example in EPIC-Oxford and UK Biobank, showed many highly statistically significant differences between diet groups in the expected directions [8, 54, 57]. The resurvey data showed that the great majority of vegetarians maintained their diet at follow-up and only a minority changed dietary group, which would be expected to result in some attenuation of risk estimates towards the null. All the main analyses were adjusted for BMI but not for energy intake, because the relationship of energy intake with cancer risk is through adiposity, which is approximated by BMI [58]. Our findings may also be affected by residual confounding due to non-dietary factors; the associations of vegetarian diets with major cancer risk factors were broadly similar across the cohorts, with vegetarians generally having a healthier profile including lower levels of smoking and alcohol consumption, and while we adjusted for these and other potential confounders there may still be some confounding due to missing data and imprecisely measured or unmeasured risk factors, such as family history of cancer, as well as possible differences between diet groups in cancer screening.

## Conclusion

This consortium includes the great majority of prospective data currently available worldwide on vegetarian diets and cancer risk. Among the people studied, most of whom lived in the UK or USA, poultry eaters had a lower risk of prostate cancer, pescatarian diets were associated with lower risks of colorectal, breast and kidney cancer, vegetarian diets were associated with lower risks of cancers of the pancreas, breast, prostate, kidney and multiple myeloma but a higher risk of squamous cell carcinoma of the oesophagus, and vegan diets were associated with a higher risk of colorectal cancer although the number of cases among vegans was small. Future research should examine the possible mediating roles of both metabolic factors and nutritional deficiencies, and collect more data particularly in vegans and in populations outside Western Europe and North America. The generalisability of the findings should be considered cautiously, because the diets and nutritional intakes of both vegetarians and non-vegetarians can vary substantially within and between populations.

## Supplementary information

Vegetarian diets and cancer risk: pooled analysis of 1.8 million women and men in nine prospective studies on three continents. Supplementary materials

Vegetarian diets and cancer risk: pooled analysis of 1.8 million women and men in nine prospective studies on three continents. Supplementary Table 3

Strobe statement

## Data Availability

The individual participant data are owned by the individual participating cohorts and are available to researchers on consent from participating cohorts.

## Code availability

Code available through contact with the research team.
