# Correction: Vegetarian diets and cancer risk: pooled analysis of 1.8 million women and men in nine prospective studies on three continents

**DOI:** 10.1038/s41416-026-03446-6

**Published:** 2026-04-13

**Authors:** Yashvee Dunneram, Jia Yi Lee, Cody Z. Watling, Izabella Lawson, Mahboubeh Parsaeian, Gary E. Fraser, Fayth M. Butler, Dorairaj Prabhakaran, Krithiga Shridhar, Dimple Kondal, Viswanathan Mohan, Mohammed K. Ali, K. M. Venkat Narayan, Nikhil Tandon, Tammy Y. N. Tong, Ruth C. Travis, Tina H. T. Chiu, Ming-Nan Lin, Chin-Lon Lin, Hsin-Chou Yang, Yu-Jen Liang, Darren C. Greenwood, Gillian K. Reeves, Keren Papier, Sarah Floud, Rashmi Sinha, Linda M. Liao, Erikka Loftfield, Janet E. Cade, Timothy J. Key, Aurora Perez-Cornago

**Affiliations:** 1 Cancer Epidemiology Unit, Nuffield Department of Population Health, University of Oxford, Oxford, UK; 2 Human Nutrition and Exercise Research Centre, Faculty of Medical Sciences, Newcastle University, Newcastle upon Tyne, UK; 3 MRC Epidemiology Unit, University of Cambridge School of Clinical Medicine, Cambridge, UK; 4 Division of Cancer Epidemiology and Genetics, National Cancer Institute, Bethesda, MD USA; 5 Centre for Nutrition, Healthy Lifestyles and Disease Prevention, School of Public Health, Loma Linda University, Loma Linda, CA USA; 6 Centre for Chronic Disease Control, New Delhi, India; 7 Emory Global Diabetes Research Center, Woodruff Health Sciences Center and Emory University, Atlanta, GA USA; 8 London School of Hygiene and Tropical Medicine, London, UK; 9 Centre for Health Analytics, Research, and Trends, Trivedi School of Bioscience, Ashoka University, Sonipat, Haryana India; 10 Madras Diabetes Research Foundation (ICMR Collaborating Centre of Excellence) and Dr. Mohan’s Diabetes Specialities Centre (IDF Centre of Excellence in Diabetes Care), Chennai, India; 11 Hubert Department of Global Health, Emory University, Atlanta, GA USA; 12 Department of Epidemiology, Rollins School of Public Health, Emory University, Atlanta, GA USA; 13 Department of Family and Preventive Medicine, School of Medicine, Emory University, Atlanta, GA USA; 14 Department of Endocrinology and Metabolism, All India Institute of Medical Sciences, New Delhi, India; 15 Department of Nutritional Science, Fu-Jen Catholic University, New Taipei City, Taiwan; 16 National Center for Geriatrics and Welfare Research, National Health Research Institutes, Yunlin, Taiwan; 17 Department of Family Medicine, Dalin Tzu Chi Hospital, Buddhist Tzu Chi Medical Foundation, Chiayi, Taiwan; 18 Department of Family Medicine, College of Medicine, Tzu Chi University, Hualien, Taiwan; 19 Buddhist Tzu Chi Medical Foundation, Hualien, Taiwan; 20 Institute of Statistical Science, Academia Sinica, Taipei, Taiwan; 21 School of Medicine, University of Leeds, Leeds, UK; 22 Nutritional Epidemiology Group, School of Food Science and Nutrition, University of Leeds, Leeds, UK

**Keywords:** Epidemiology, Cancer epidemiology

Correction to: *British Journal of Cancer* 10.1038/s41416-025-03327-4, published online 27 February 2026

Following publication of the article, errors were found in the text which required correction.

‘METHODS – Statistical analyses’, final paragraph originally read: “FDR, among the 16 HRs shown in the main Figs. 1 to 3”. This should instead read: “FDR, among the 16 cancer sites shown in the main Figs. 1 to 3”.

‘RESULTS – Cancers of the reproductive system’ originally read: “…and in vegetarians (0.91 (0.86 to 0.97))”. This should instead read: “…and in vegetarians (0.91 (0.86 to 0.97, FDR significant))".

The original article has been corrected.

