# Supplementary material for: Vegetarian diets and cancer risk: pooled analysis of 1.8 million women and men in nine prospective studies on three continents
Source: Br J Cancer. 2026 Feb 27;134(8):1218–29. doi: 10.1038/s41416-025-03327-4 (PMC13035945; doi:10.1038/s41416-025-03327-4)
Supplement: Supplementary file 1 — Vegetarian diets and cancer risk: pooled analysis of 1.8 million women and men in nine prospective studies on three continents. Supplementary materials [file 41416_2025_3327_MOESM1_ESM.docx]

**Vegetarian diets and cancer risk: pooled analysis of 1.8 million women and men in nine prospective studies on three continents**

Contents

[Supplementary Methods 3](#_Toc176187422)

[Supplementary Tables 5](#_Toc176187423)

[Supplementary Table 1. Method of cancer outcome ascertainment in each cohort 5](#_Toc176187424)

[Supplementary Table 2. Percentage of missing or unknown covariates in each cohort 6](#_Toc176187425)

[Supplementary Table 3. Risks for 17 cancer sites compared to meat eaters: overall, without adjustment for BMI, excluding the first four years of follow up, and in never smokers 7](#_Toc176187426)

[Supplementary Table 4: Pooled hazard ratios for breast cancer by diet group in premenopausal and postmenopausal women 8](#_Toc176187427)

[Supplementary Figures 9](#_Toc176187428)

[Supplementary Figure 1. Study-specific and pooled multivariable-adjusted hazard ratios (HRs) and 95% confidence intervals comparing the risk of mouth and pharynx cancer in poultry eaters, pescatarians and vegetarians to that in meat eaters; no results shown for vegans because there were fewer than 10 cases. 9](#_Toc176187429)

[Supplementary Figure 2. Study-specific and pooled multivariable-adjusted hazard ratios (HRs) and 95% confidence intervals comparing the risk of oesophageal squamous cell carcinoma in poultry eaters, pescatarians, vegetarians to that in meat eaters; no results shown for vegans because there were fewer than 10 cases. 10](#_Toc176187430)

[Supplementary Figure 3. Study-specific and pooled multivariable-adjusted hazard ratios (HRs) and 95% confidence intervals comparing the risk of oesophageal adenocarcinoma in poultry eaters, pescatarians, vegetarians to that in meat eaters; no results shown for vegans because there were fewer than 10 cases. 11](#_Toc176187431)

[Supplementary Figure 4 Study-specific and pooled multivariable-adjusted hazard ratios (HRs) and 95% confidence intervals comparing the risk of stomach cancer in poultry eaters, pescatarians, vegetarians to that in meat eaters; no results shown for vegans because there were fewer than 10 cases. 12](#_Toc176187432)

[Supplementary Figure 5. Study-specific and pooled multivariable-adjusted hazard ratios (HRs) and 95% confidence intervals comparing the risk of colorectal cancer in poultry eaters, pescatarians, vegetarians, and vegans to that in meat eaters. 13](#_Toc176187433)

[Supplementary Figure 6. Study-specific and pooled multivariable-adjusted hazard ratios (HRs) and 95% confidence intervals comparing the risk of liver cancer in poultry eaters, pescatarians and vegetarians to that in meat eaters; no results shown for vegans because there were fewer than 10 cases. 14](#_Toc176187434)

[Supplementary Figure 7. Study-specific and pooled multivariable-adjusted hazard ratios (HRs) and 95% confidence intervals comparing the risk of pancreatic cancer in poultry eaters, pescatarians, vegetarians, and vegans to that in meat eaters. 15](#_Toc176187435)

[Supplementary Figure 8. Study-specific and pooled multivariable-adjusted hazard ratios (HRs) and 95% confidence intervals comparing the risk of lung cancer (never smokers) in poultry eaters, pescatarians, vegetarians, and vegans to that in meat eaters. 16](#_Toc176187436)

[Supplementary Figure 9. Study-specific and pooled multivariable-adjusted hazard ratios (HRs) and 95% confidence intervals comparing the risk of breast cancer in poultry eaters, pescatarians, vegetarians, and vegans to that in meat eaters. 17](#_Toc176187437)

[Supplementary Figure 10. Study-specific and pooled multivariable-adjusted hazard ratios (HRs) and 95% confidence intervals comparing the risk of endometrial cancer in poultry eaters, pescatarians, vegetarians, and vegans to that in meat eaters. 18](#_Toc176187438)

[Supplementary Figure 11. Study-specific and pooled multivariable-adjusted hazard ratios (HRs) and 95% confidence intervals comparing the risk of ovarian cancer in poultry eaters, pescatarians, vegetarians, and vegans to that in meat eaters. 19](#_Toc176187439)

[Supplementary Figure 12. Study-specific and pooled multivariable-adjusted hazard ratios (HRs) and 95% confidence intervals comparing the risk of prostate cancer in poultry eaters, pescatarians, vegetarians, and vegans to that in meat eaters. 20](#_Toc176187440)

[Supplementary Figure 13. Study-specific and pooled multivariable-adjusted hazard ratios (HRs) and 95% confidence intervals comparing the risk of kidney cancer in poultry eaters, pescatarians and vegetarians to that in meat eaters; no results shown for vegans because there were fewer than 10 cases. 21](#_Toc176187441)

[Supplementary Figure 14. Study-specific and pooled multivariable-adjusted hazard ratios (HRs) and 95% confidence intervals comparing the risk of bladder cancer in poultry eaters, pescatarians, vegetarians, and vegans to that in meat eaters. 22](#_Toc176187442)

[Supplementary Figure 15. Study-specific and pooled multivariable-adjusted hazard ratios (HRs) and 95% confidence intervals comparing the risk of non-Hodgkin lymphoma in poultry eaters, pescatarians, vegetarians, and vegans to that in meat eaters. 23](#_Toc176187443)

[Supplementary Figure 16. Study-specific and pooled multivariable-adjusted hazard ratios (HRs) and 95% confidence intervals comparing the risk of multiple myeloma in poultry eaters, pescatarians, vegetarians, and vegans to that in meat eaters. 24](#_Toc176187444)

[Supplementary Figure 17. Study-specific and pooled multivariable-adjusted hazard ratios (HRs) and 95% confidence intervals comparing the risk of leukaemia in poultry eaters, pescatarians, vegetarians, and vegans to that in meat eaters. 25](#_Toc176187445)

# Supplementary Methods

*Covariate classification.* The models were stratified by sex (where appropriate), and by region or method of recruitment (CARRS-1: Chennai, Delhi; EPIC-Oxford: postal recruitment, general practitioner recruitment; Million Women Study: Oxford, East Anglia, South West, Thames, West Midlands, Yorkshire, Trent, North West (Mersey), North West Manchester and Lancashire, Scotland; UK Biobank: North-West England, North-East England, Yorkshire and the Humber, West Midlands, East Midlands, South-East England, South-West England, London, Wales, Scotland).

Covariates in the multivariable-adjusted models were: living with a partner (yes, no), educational status (less than secondary/high school, secondary/high school or equivalent, university degree or equivalent), ethnic group (Asian, Black, Hispanic, White, other), study and sex-specific height categories (women in UK and USA cohorts: <160, 160-164.9, ≥165 cm; women in Asian cohorts: <150, 150-154.9, ≥155 cm; men in UK and USA cohorts: <175, 175-179.9, ≥180 cm; men in Asian cohorts: <163, 163-167.9, ≥168 cm;), cigarette smoking (never, previous, current <10 cigarettes/day, current 10-19 cigarettes/day, current ≥20 cigarettes/day, current unknown number of cigarettes), tobacco chewing (in CARRS-1 only; never, previous, current), physical activity (highly active, moderately active, inactive), alcohol intake (zero, >0-9.9, 10.0-19.9, ≥20.0 g/day), history of diabetes (yes, no), parity (nulliparous, parous), ever used hormone replacement therapy (yes, no), and BMI (<20.0, 20.0-22.4, 22.5-24.9, 25.0-29.9, ≥30.0 kg/m^2^). For breast, endometrial, and ovarian cancers, the models were further adjusted for age at menarche (≤10 years, 11-12 years, 13-14 years, ≥15 years), parity and age at first birth combined (nulliparous, and parity and age at first birth grouped as: 1-2 and <25, 1-2 and 25-29, 1-2 and ≥30, 1-2 and unknown, ≥3 and <25, ≥3 and 25-29, ≥3 and ≥30, ≥3 and unknown), menopausal status (pre-menopausal, post-menopausal), and ever used oral contraceptives (yes, no). For prostate cancer, we further adjusted for history of prostate specific antigen screening (yes, no). For all the covariates, missing or unknown data were categorised separately as unknown (percentages missing or unknown for each covariate, in each cohort, are shown in supplementary table 2).

# Supplementary Tables

## Supplementary Table 1. Method of cancer outcome ascertainment in each cohort

| **Cohort** | **Cancer ascertainment** | **Incident cancer definition** |
| --- | --- | --- |
| ***Cohorts with large numbers of vegetarians*** | |  |
| Adventist Health Study-2 | Linkage to 32 state cancer registries | ICD-O-3 |
| Center for cArdiometabolic Risk Reduction in South Asia-1 | Linkage to cancer registry/self-report/verbal autopsy | ICD-10 |
| European Prospective Investigation into Cancer and Nutrition-Oxford | Linkage to the UK National Health Service | ICD-9, ICD-10 |
| Oxford Vegetarian Study | Linkage to the UK National Health Service | ICD-9, ICD-10 |
| Tzu Chi Health Study | Linkage to the National Cancer Registry and National Mortality Registry | ICD-9 for cancer registry, ICD-10 for mortality registry |
| UK Women's Cohort Study | Linkage to the UK National Health Service | ICD-9, ICD-10 |
| ***Very large cohort studies*** | | |
| Million Women Study | Linkage to the UK National Health Service | ICD-10 |
| National Institutes of Health-AARP Diet and Health Study | Linkage to eleven state cancer registries (California, Florida, Louisiana, New Jersey, North Carolina, Michigan, Georgia, Pennsylvania, Arizona, Nevada and Texas) | ICD-O-3 |
| UK Biobank | Linkage to the UK National Health Service | ICD-9, ICD-10 |

Abbreviation: ICD, International Classification of Diseases.

## Supplementary Table 2. Percentage of missing or unknown covariates in each cohort

| **Variable** | **AHS-2** | **CARRS-1** | **EPIC-Oxford** | **OVS** | **TCHS** | **UKWCS** | **MWS** | **NIH-AARP** | **UK Biobank** | |  |
| --- | --- | --- | --- | --- | --- | --- | --- | --- | --- | --- | --- |
| Height | 1.7 | 19.9 | 1.9 | <1 | 0 | 2.1 | 1 | 1.1 | <1 | |  |
| Living with partner | 1.7 | 0 | <1 | <1 | <1 | 1.5 | 1.4 | <1 | 19 | |  |
| Educational status | 1.3 | 0 | 7.4 | 18.4 | 0 | 8.5 | 1.6 | 2.8 | 1.8 | |  |
| Ethnic group | 0 | 0 | 2.2 | 0 | 0 | 2.7 | 1.1 | 1.2 | <1 | |  |
| Smoking status and number of cigarettes | 1.4 | 6.6 | <1 | <1 | 0 | 3.1 | 1.4 | 3.7 | <1 | |  |
| Physical activity | 4.8 | 22.9 | 13.9 | 2.1 | 0 | <1 | 26 | 1 | 3.9 | |  |
| Alcohol intake (g/day) | 0 | - | 0 | 0 | <1 | 0 | 0 | 0 | 0 | |  |
| History of diabetes | 1.2 | <1 | 8.7 | <1 | 0 | 7.7 | 0 | 0 | <1 | |  |
| Parity and age at first birth | 1.4 | <1 | <1 | 2.4 | <1^1^ | 10.4 | <1 | 1.1 | <1 | |  |
| Ever used hormone replacement therapy | 3 | - | 1.4 | - | <1^2^ | 3.2 | 1.4 | 0 | <1 | |  |
| Body mass index | 2.5 | 22.9 | 3.3 | 1.6 | 0 | 3.7 | 1.7 | 2.4 | <1 | |  |
| Age at menarche | <1 | <1 | 1.4 | - | <1 | 2 | 1.5 | <1 | 3.1 | |  |
| Ever used oral contraceptives | 0 | <1 | <1 | <1 | <1 | 1.5 | <1 | 1.5 | <1 | |  |
| History of prostate cancer screening | 9.8 | - | - | - | NA | NA | NA | 43.5 | 5.4 | |  |
| ^1^<1% missing for parity; age at first birth not asked.  ^2^Among postmenopausal women (this question not answered by most premenopausal women).  - indicates that this variable was not available in the specific cohort.  Abbreviations: AHS-2, Adventist Health Study-2; CARRS, Center for cArdiometabolic Risk Reduction in South Asia; EPIC, European Prospective Investigation into Cancer and Nutrition; MWS, Million Women Study; NA, not applicable; NIH-AARP, National Institutes of Health-AARP Diet and Health Study; OVS, Oxford Vegetarian Study; TCHS, Tzu Chi Health Study; UKWCS, UK Women’s Cohort Study. | | | | | | | | | |  | |

## Supplementary Table 3. Risks for 17 cancer sites compared to meat eaters: overall, without adjustment for BMI, excluding the first four years of follow up, and in never smokers

Please see separate file

## Supplementary Table 4: Pooled hazard ratios for breast cancer by diet group in premenopausal and postmenopausal women

|  | **Premenopausal** | | **Postmenopausal** | |
| --- | --- | --- | --- | --- |
| **Diet group** | **Cases** | **HR (95% CI)** | **Cases** | **HR (95% CI)** |
| Meat eaters | 1674 | Reference | 54,957 | Reference |
| Poultry eaters | 56 | 1.01 (0.76 to 1.33) | 1605 | 0.96 (0.91 to 1.01) |
| Pescatarians | 234 | 1.03 (0.89 to 1.20) | 1165 | 0.91 (0.86 to 0.97) |
| Vegetarians | 353 | 1.00 (0.87 to 1.15) | 1114 | 0.89 (0.83 to 0.95) |
| Vegans | 27 | 0.85 (0.57 to 1.27) | 90 | 0.86 (0.69 to 1.07) |
| Premenopausal categorised as women premenopausal at recruitment and breast cancers diagnosed before age 55.  Postmenopausal categorised as women classed as postmenopausal at recruitment, and all breast cancers diagnosed at ages 55 and above.  Tzu Chi Health Study not included.  Abbreviations: CI, confidence intervals; HR, hazard ratio. | | | | |

# Supplementary Figures

##
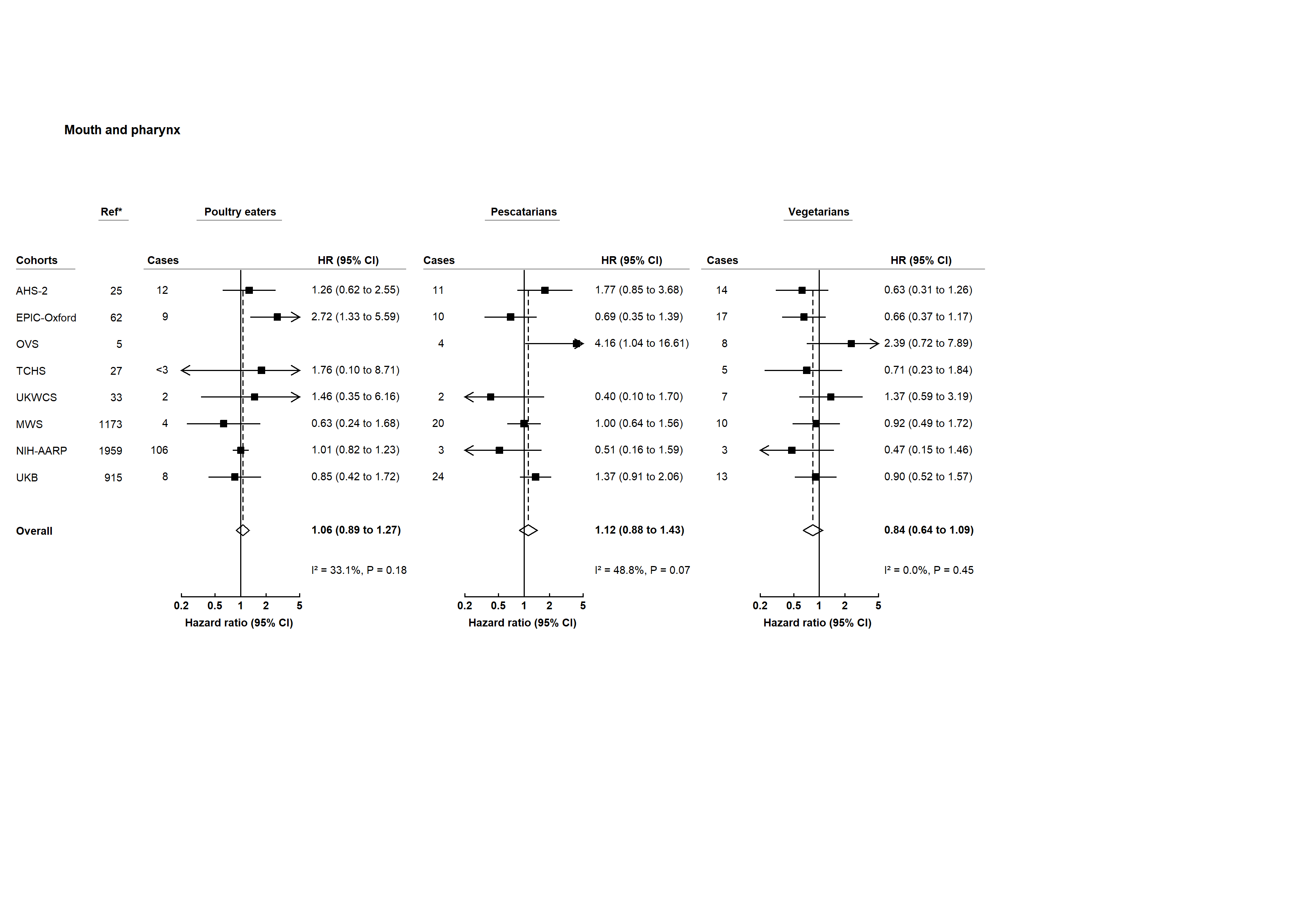


## Supplementary Figure 1. Study-specific and pooled multivariable-adjusted hazard ratios (HRs) and 95% confidence intervals comparing the risk of mouth and pharynx cancer in poultry eaters, pescatarians and vegetarians to that in meat eaters.

No results are shown for vegans because there were fewer than 10 cases across all cohorts. All models used age as the underlying time variable and are stratified by region or method of recruitment and sex, and adjusted for living with partner, educational status, ethnic group, height, body mass index, cigarette smoking, physical activity, alcohol intake, history of diabetes, parity, and ever use of hormone replacement therapy. Confidence intervals extending beyond the axis are marked with arrows.

*Ref, number of cases in the reference group of meat eaters.

Abbreviations: AHS-2, Adventist Health Study-2; EPIC, European Prospective Investigation into Cancer and Nutrition; MWS, Million Women Study; NIH-AARP, National Institutes of Health-AARP Diet and Health Study; OVS, Oxford Vegetarian Study; TCHS, Tzu Chi Health Study; UKWCS, UK Women’s Cohort Study; UKB, UK Biobank.


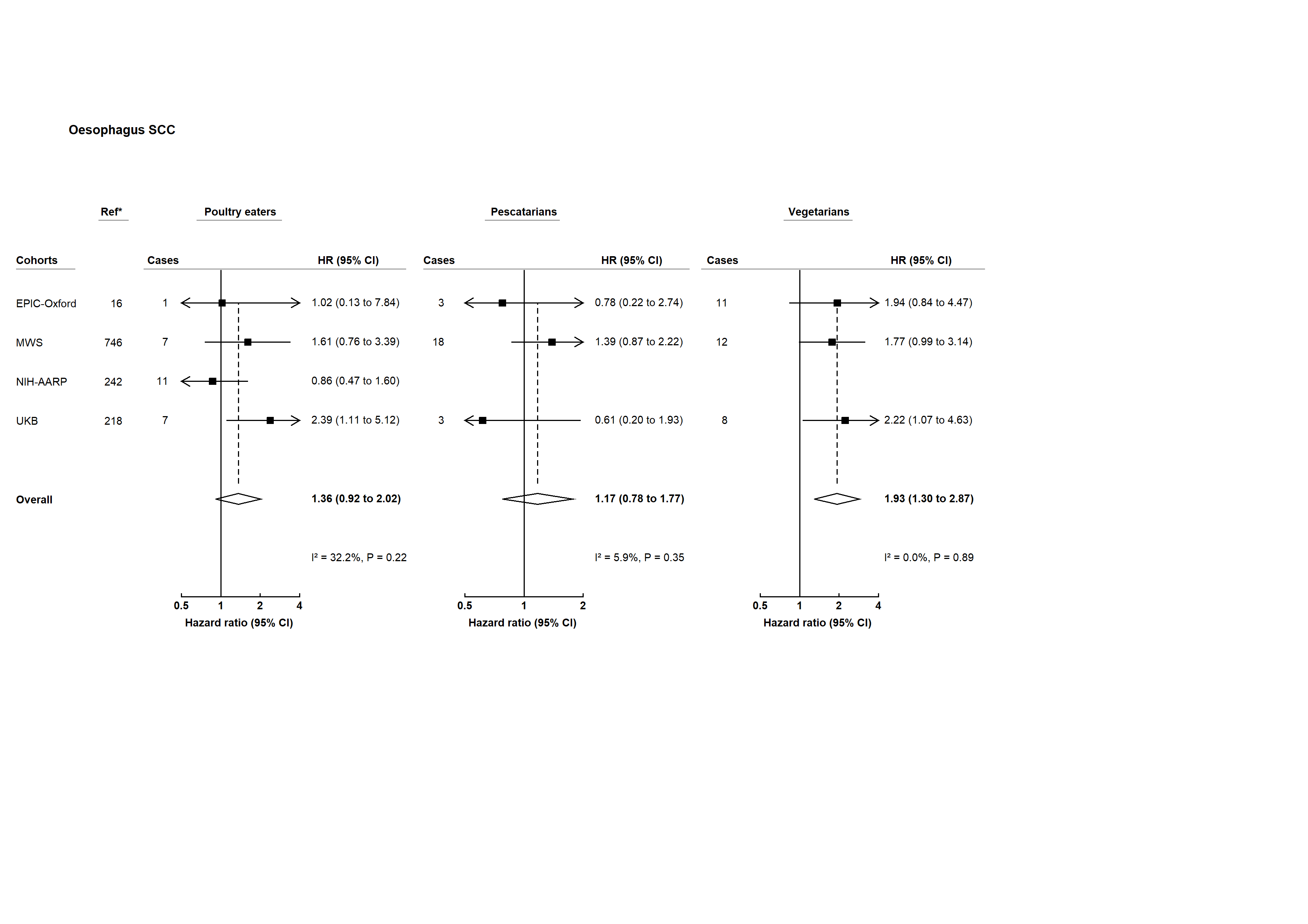


## Supplementary Figure 2. Study-specific and pooled multivariable-adjusted hazard ratios (HRs) and 95% confidence intervals comparing the risk of oesophageal squamous cell carcinoma in poultry eaters, pescatarians and vegetarians to that in meat eaters.

No results are shown for vegans because there were fewer than 10 cases across all cohorts. All models used age as the underlying time variable and are stratified by region or method of recruitment and sex, and adjusted for living with partner, educational status, ethnic group, height, body mass index, cigarette smoking, physical activity, alcohol intake, history of diabetes, parity, and ever use of hormone replacement therapy. Confidence intervals extending beyond the axis are marked with arrows.

*Ref, number of cases in the reference group of meat eaters.

Abbreviations: EPIC, European Prospective Investigation into Cancer and Nutrition; MWS, Million Women Study; NIH-AARP, National Institutes of Health-AARP Diet and Health Study; UKB, UK Biobank.


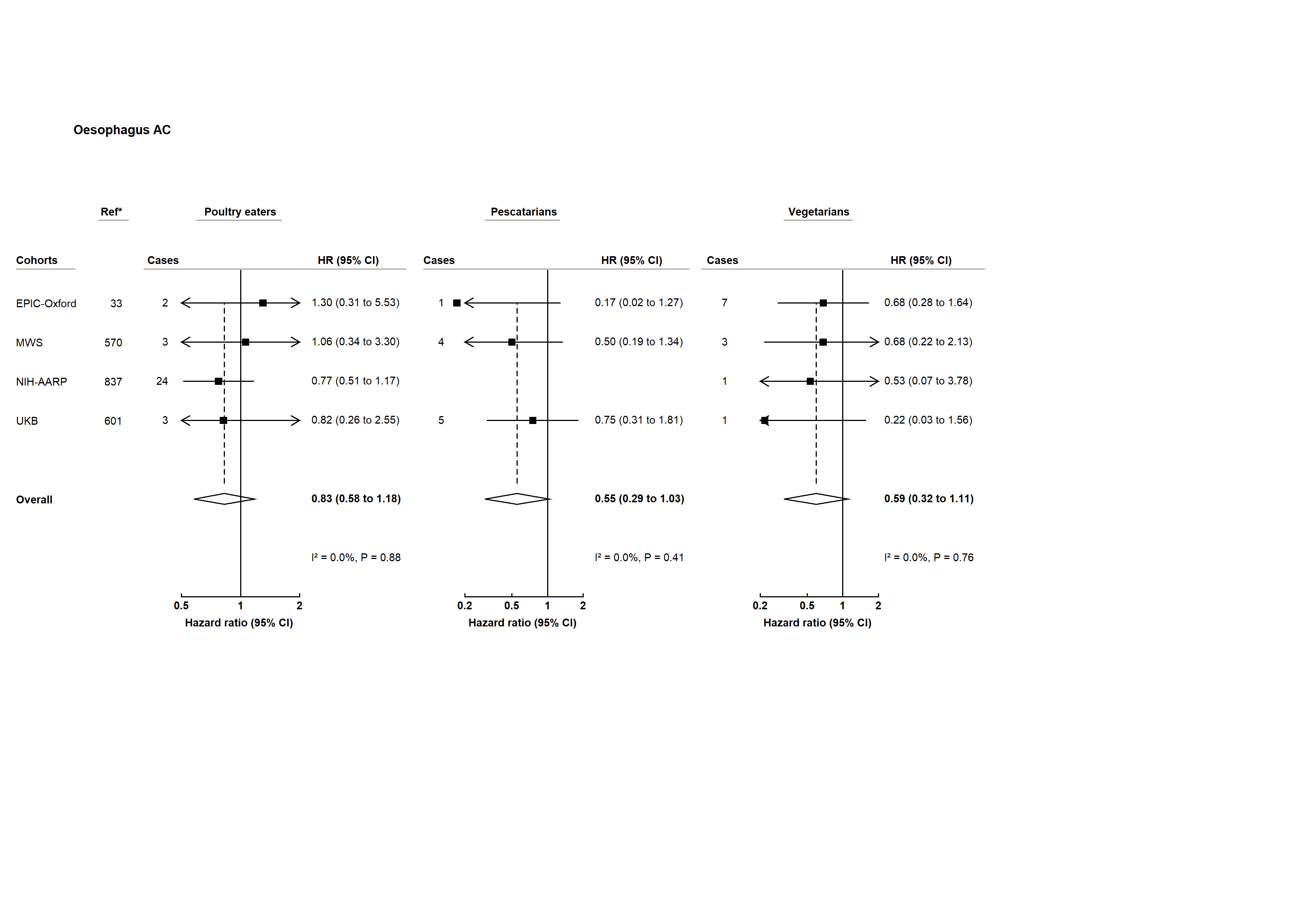


## Supplementary Figure 3. Study-specific and pooled multivariable-adjusted hazard ratios (HRs) and 95% confidence intervals comparing the risk of oesophageal adenocarcinoma in poultry eaters, pescatarians and vegetarians to that in meat eaters.

No results are shown for vegans because there were fewer than 10 cases across all cohorts. All models used age as the underlying time variable and are stratified by region or method of recruitment and sex, and adjusted for living with partner, educational status, ethnic group, height, body mass index, cigarette smoking, physical activity, alcohol intake, history of diabetes, parity, and ever use of hormone replacement therapy. Confidence intervals extending beyond the axis are marked with arrows.

*Ref, number of cases in the reference group of meat eaters.

Abbreviations: EPIC, European Prospective Investigation into Cancer and Nutrition; MWS, Million Women Study; NIH-AARP, National Institutes of Health-AARP Diet and Health Study; UKB, UK Biobank.


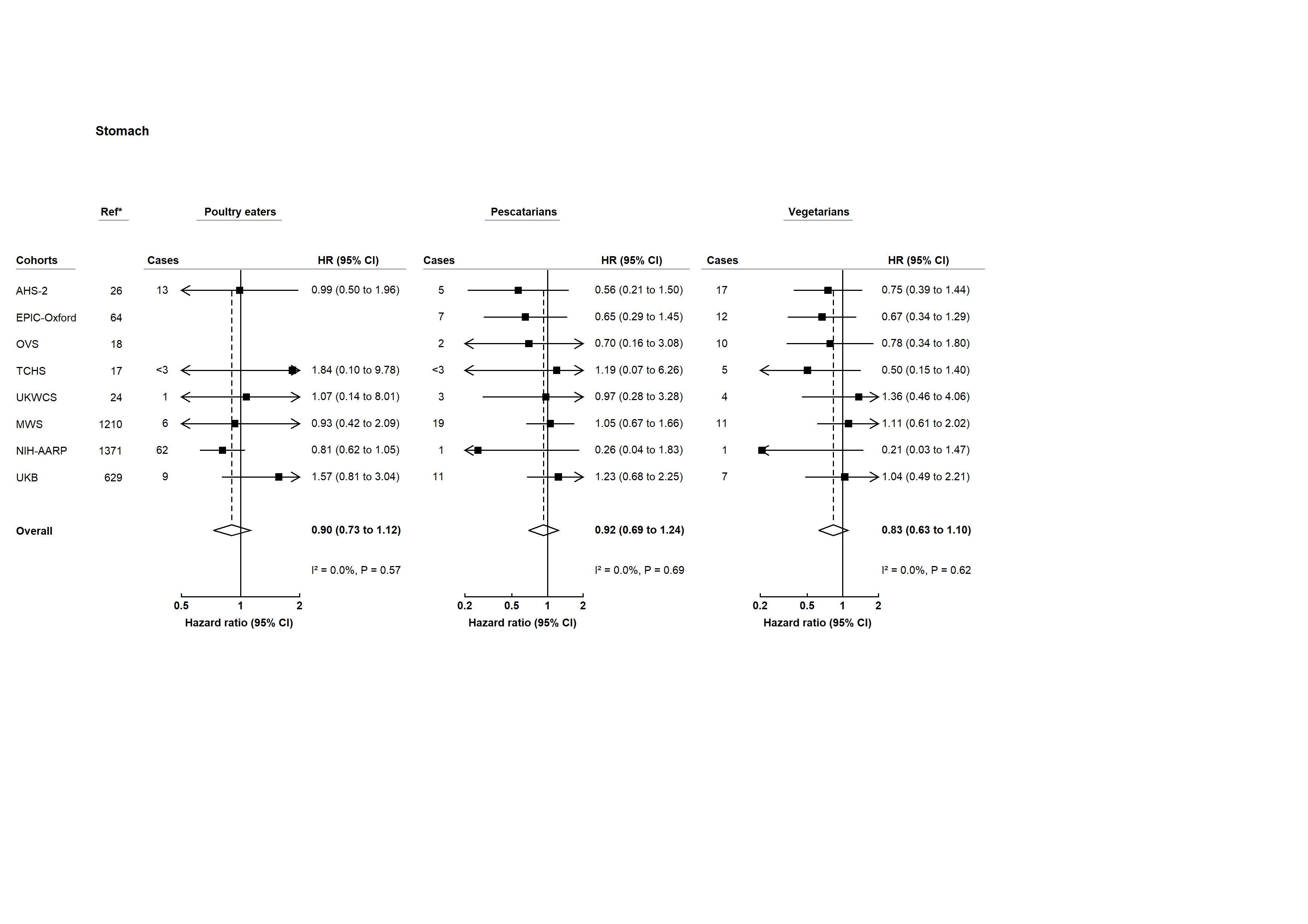


## Supplementary Figure 4. Study-specific and pooled multivariable-adjusted hazard ratios (HRs) and 95% confidence intervals comparing the risk of stomach cancer in poultry eaters, pescatarians and vegetarians to that in meat eaters.

No results are shown for vegans because there were fewer than 10 cases across all cohorts. All models used age as the underlying time variable and are stratified by region or method of recruitment and sex, and adjusted for living with partner, educational status, ethnic group, height, body mass index, cigarette smoking, physical activity, alcohol intake, history of diabetes, parity, and ever use of hormone replacement therapy. Confidence intervals extending beyond the axis are marked with arrows.

*Ref, number of cases in the reference group of meat eaters.

Abbreviations: AHS-2, Adventist Health Study-2; EPIC, European Prospective Investigation into Cancer and Nutrition; MWS, Million Women Study; NIH-AARP, National Institutes of Health-AARP Diet and Health Study; OVS, Oxford Vegetarian Study; TCHS, Tzu Chi Health Study; UKWCS, UK Women’s Cohort Study; UKB, UK Biobank.


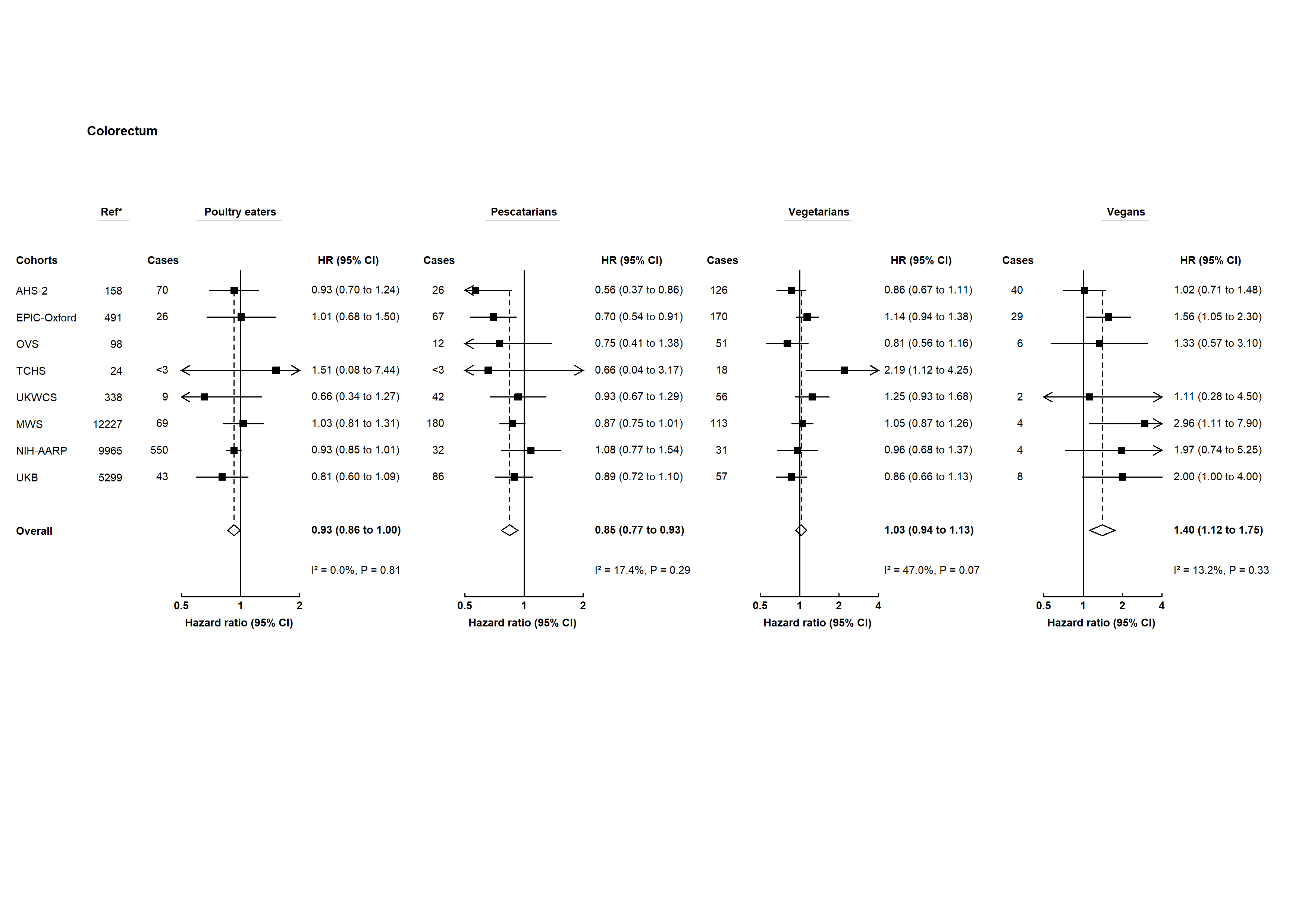


## Supplementary Figure 5. Study-specific and pooled multivariable-adjusted hazard ratios (HRs) and 95% confidence intervals comparing the risk of colorectal cancer in poultry eaters, pescatarians and vegetarians to that in meat eaters.

All models used age as the underlying time variable and are stratified by region or method of recruitment and sex, and adjusted for living with partner, educational status, ethnic group, height, body mass index, cigarette smoking, physical activity, alcohol intake, history of diabetes, parity, and ever use of hormone replacement therapy. Confidence intervals extending beyond the axis are marked with arrows.

*Ref, number of cases in the reference group of meat eaters.

Abbreviations: AHS-2, Adventist Health Study-2; EPIC, European Prospective Investigation into Cancer and Nutrition; MWS, Million Women Study; NIH-AARP, National Institutes of Health-AARP Diet and Health Study; OVS, Oxford Vegetarian Study; TCHS, Tzu Chi Health Study; UKWCS, UK Women’s Cohort Study; UKB, UK Biobank.


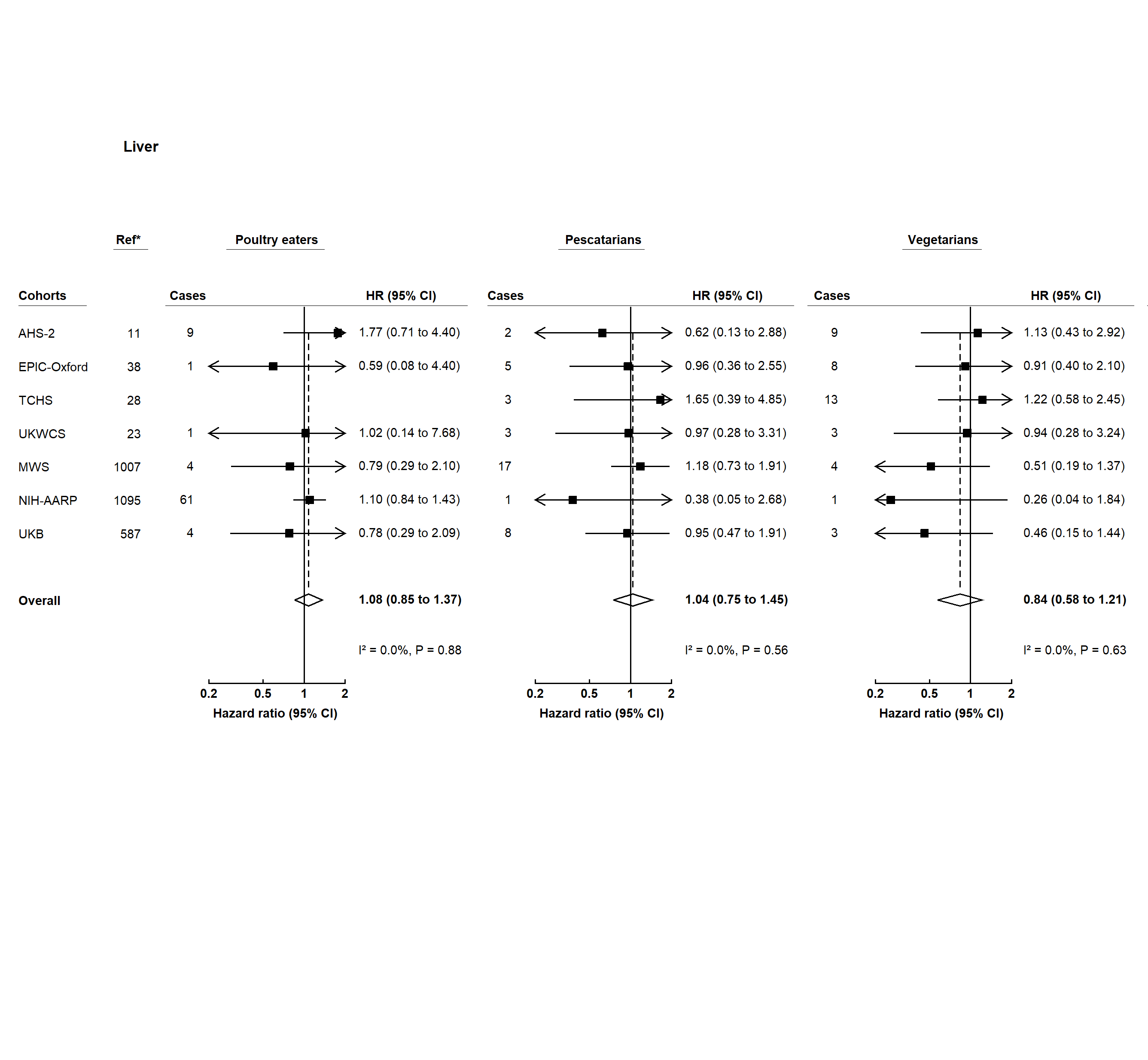


## Supplementary Figure 6. Study-specific and pooled multivariable-adjusted hazard ratios (HRs) and 95% confidence intervals comparing the risk of liver cancer in poultry eaters, pescatarians and vegetarians to that in meat eaters.

No results are shown for vegans because there were fewer than 10 cases across all cohorts. All models used age as the underlying time variable and are stratified by region or method of recruitment and sex, and adjusted for living with partner, educational status, ethnic group, height, body mass index, cigarette smoking, physical activity, alcohol intake, history of diabetes, parity, and ever use of hormone replacement therapy. Confidence intervals extending beyond the axis are marked with arrows.

*Ref, number of cases in the reference group of meat eaters.

Abbreviations: AHS-2, Adventist Health Study-2; EPIC, European Prospective Investigation into Cancer and Nutrition; MWS, Million Women Study; NIH-AARP, National Institutes of Health-AARP Diet and Health Study; TCHS, Tzu Chi Health Study; UKWCS, UK Women’s Cohort Study; UKB, UK Biobank.


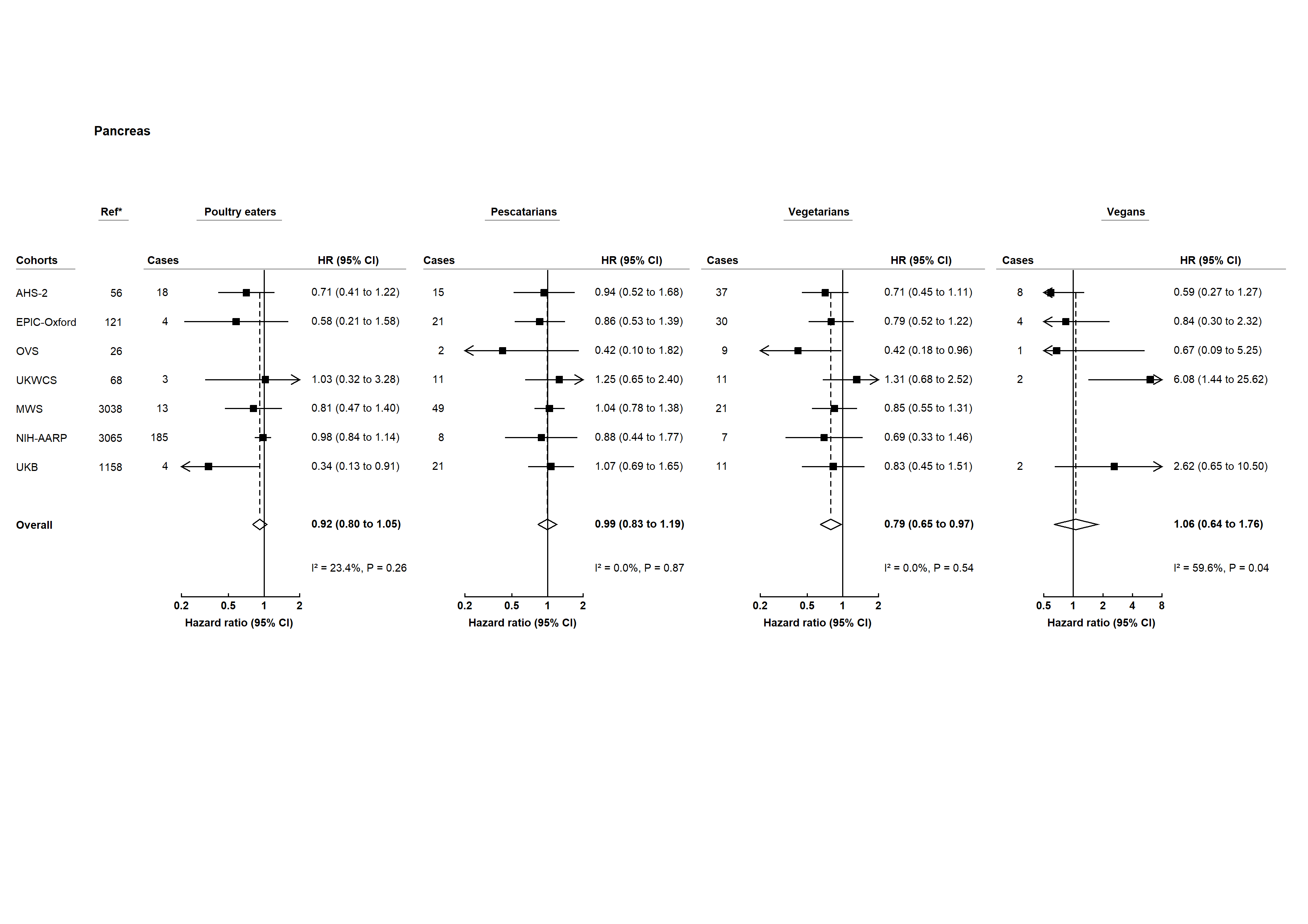


## Supplementary Figure 7. Study-specific and pooled multivariable-adjusted hazard ratios (HRs) and 95% confidence intervals comparing the risk of pancreatic cancer in poultry eaters, pescatarians and vegetarians to that in meat eaters.

All models used age as the underlying time variable and are stratified by region or method of recruitment and sex, and adjusted for living with partner, educational status, ethnic group, height, body mass index, cigarette smoking, physical activity, alcohol intake, history of diabetes, parity, and ever use of hormone replacement therapy. Confidence intervals extending beyond the axis are marked with arrows.

*Ref, number of cases in the reference group of meat eaters.

Abbreviations: AHS-2, Adventist Health Study-2; EPIC, European Prospective Investigation into Cancer and Nutrition; NIH-AARP, National Institutes of Health-AARP Diet and Health Study; OVS, Oxford Vegetarian Study; UKB, UK Biobank.


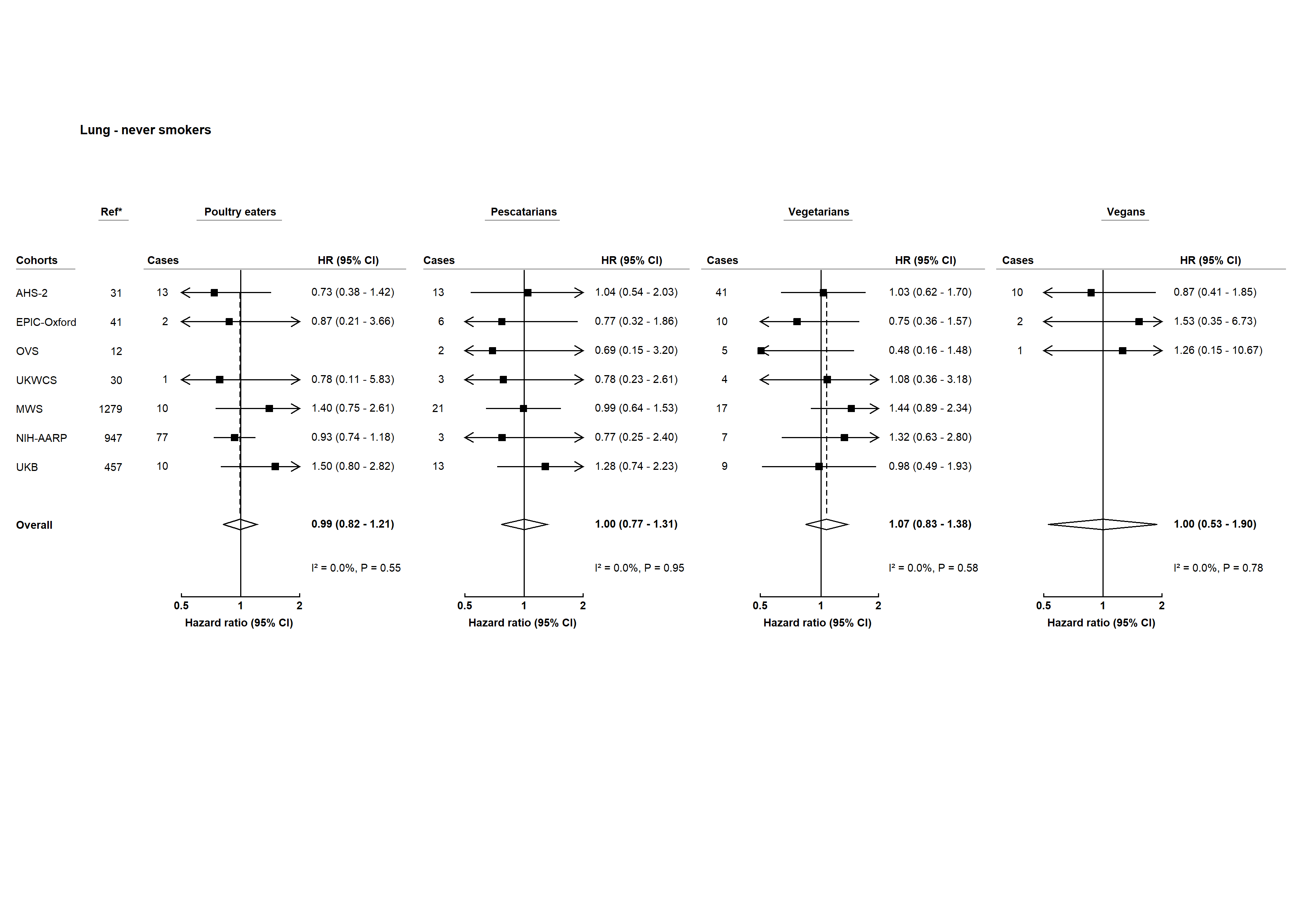


## Supplementary Figure 8. Study-specific and pooled multivariable-adjusted hazard ratios (HRs) and 95% confidence intervals comparing the risk of lung cancer (never smokers) in poultry eaters, pescatarians and vegetarians to that in meat eaters.

All models used age as the underlying time variable and are stratified by region or method of recruitment and sex, and adjusted for living with partner, educational status, ethnic group, height, body mass index, physical activity, alcohol intake, history of diabetes, parity, and ever use of hormone replacement therapy. Confidence intervals extending beyond the axis are marked with arrows.

*Ref, number of cases in the reference group of meat eaters.

Abbreviations: AHS-2, Adventist Health Study-2; EPIC, European Prospective Investigation into Cancer and Nutrition; MWS, Million Women Study; NIH-AARP, National Institutes of Health-AARP Diet and Health Study; OVS, Oxford Vegetarian Study; UKWCS, UK Women’s Cohort Study; UKB, UK Biobank.


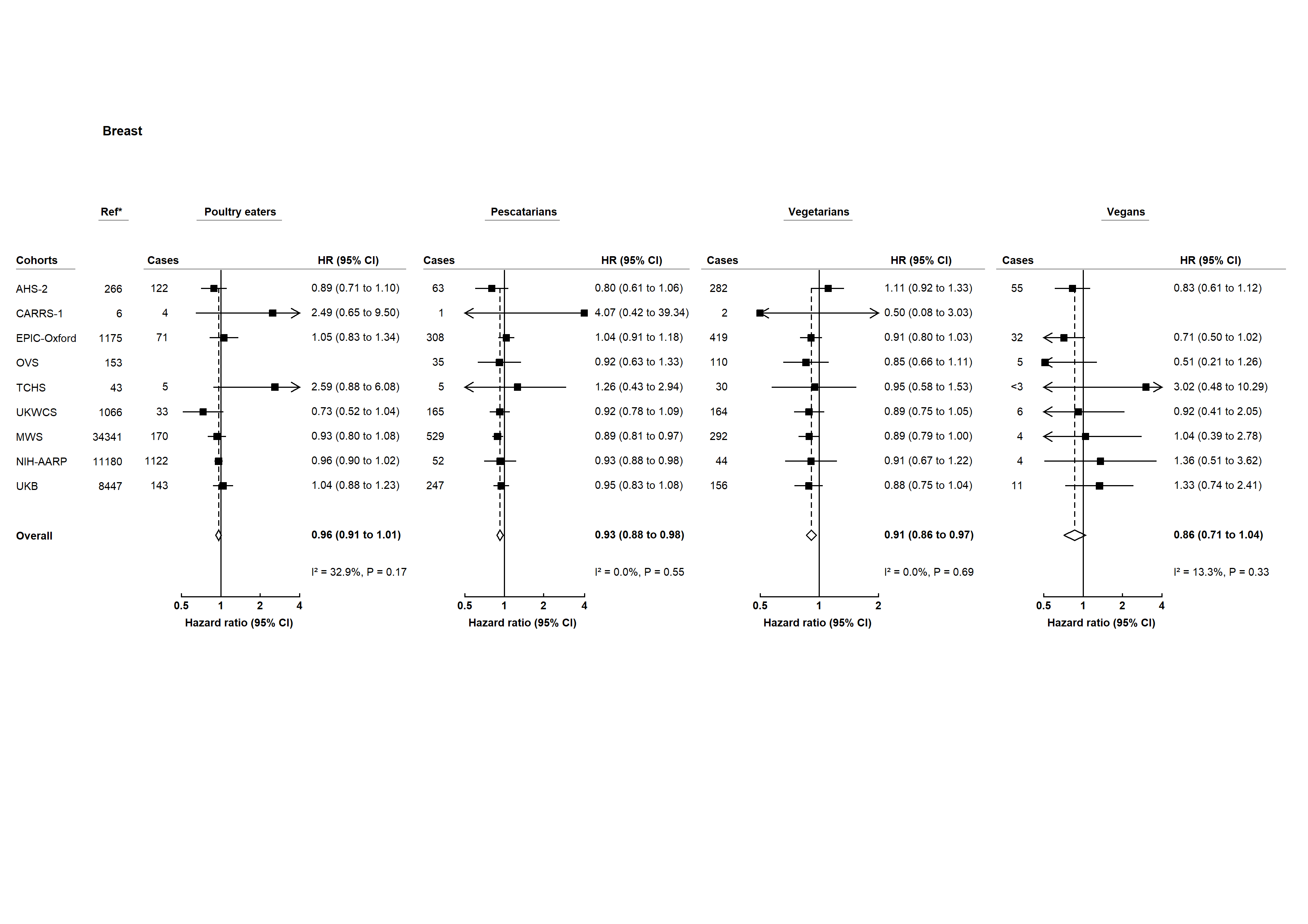


## Supplementary Figure 9. Study-specific and pooled multivariable-adjusted hazard ratios (HRs) and 95% confidence intervals comparing the risk of breast cancer in poultry eaters, pescatarians and vegetarians to that in meat eaters.

All models used age as the underlying time variable and are stratified by region or method of recruitment and sex, and adjusted for living with partner, educational status, ethnic group, height, body mass index, cigarette smoking, physical activity, alcohol intake, history of diabetes, parity and age at first birth combined, menopausal status, and ever use of hormone replacement therapy or oral contraceptives. Confidence intervals extending beyond the axis are marked with arrows.

*Ref, number of cases in the reference group of meat eaters.

Abbreviations: AHS-2, Adventist Health Study-2; CARRS, Center for cArdiometabolic Risk Reduction in South Asia; EPIC, European Prospective Investigation into Cancer and Nutrition; MWS, Million Women Study; NIH-AARP, National Institutes of Health-AARP Diet and Health Study; OVS, Oxford Vegetarian Study; TCHS, Tzu Chi Health Study; UKWCS, UK Women’s Cohort Study; UKB, UK Biobank.


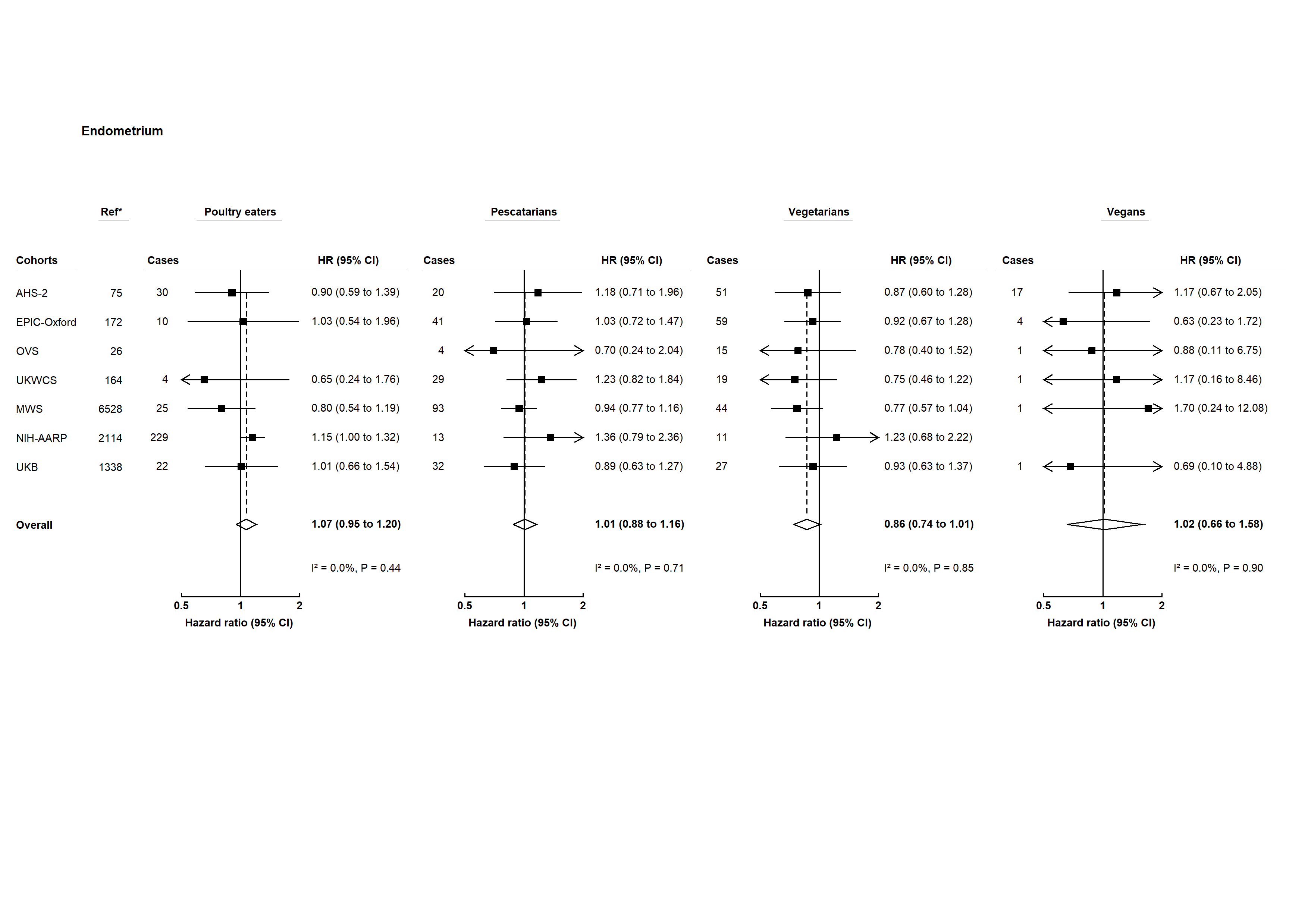


## Supplementary Figure 10. Study-specific and pooled multivariable-adjusted hazard ratios (HRs) and 95% confidence intervals comparing the risk of endometrial cancer in poultry eaters, pescatarians and vegetarians to that in meat eaters.

All models used age as the underlying time variable and are stratified by region or method of recruitment and sex, and adjusted for living with partner, educational status, ethnic group, height, body mass index, cigarette smoking, physical activity, alcohol intake, history of diabetes, parity and age at first birth combined, menopausal status, and ever use of hormone replacement therapy or oral contraceptives. Confidence intervals extending beyond the axis are marked with arrows.

*Ref, number of cases in the reference group of meat eaters.

Abbreviations: AHS-2, Adventist Health Study-2; EPIC, European Prospective Investigation into Cancer and Nutrition; MWS, Million Women Study; NIH-AARP, National Institutes of Health-AARP Diet and Health Study; OVS, Oxford Vegetarian Study; UKWCS, UK Women’s Cohort Study; UKB, UK Biobank.


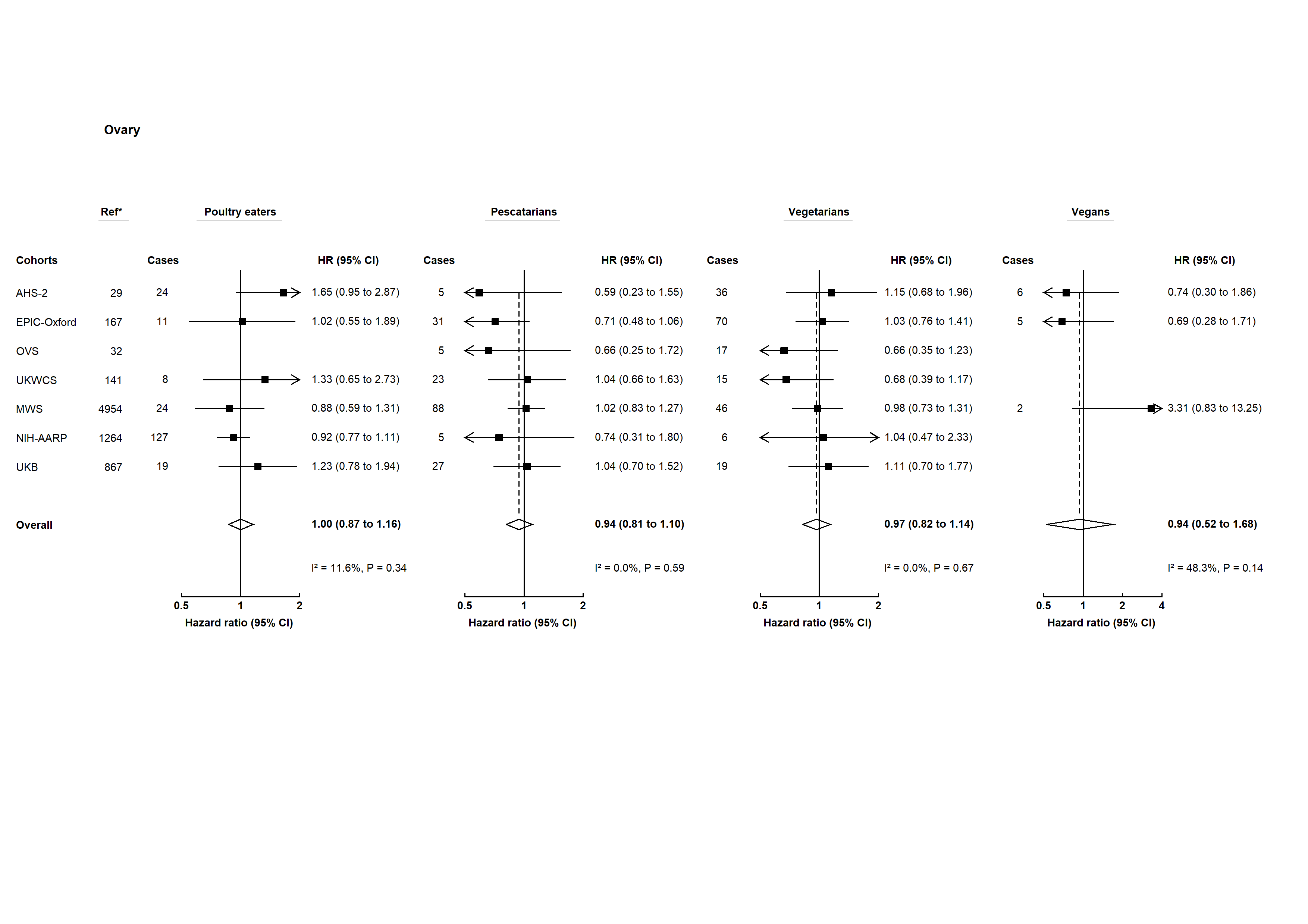


## Supplementary Figure 11. Study-specific and pooled multivariable-adjusted hazard ratios (HRs) and 95% confidence intervals comparing the risk of ovarian cancer in poultry eaters, pescatarians and vegetarians to that in meat eaters.

All models used age as the underlying time variable and are stratified by region or method of recruitment and sex, and adjusted for living with partner, educational status, ethnic group, height, body mass index, cigarette smoking, physical activity, alcohol intake, history of diabetes, parity and age at first birth combined, menopausal status, and ever use of hormone replacement therapy or oral contraceptives. Confidence intervals extending beyond the axis are marked with arrows.

*Ref, number of cases in the reference group of meat eaters.

Abbreviations: AHS-2, Adventist Health Study-2; EPIC, European Prospective Investigation into Cancer and Nutrition; MWS, Million Women Study; NIH-AARP, National Institutes of Health-AARP Diet and Health Study; OVS, Oxford Vegetarian Study; UKWCS, UK Women’s Cohort Study; UKB, UK Biobank.


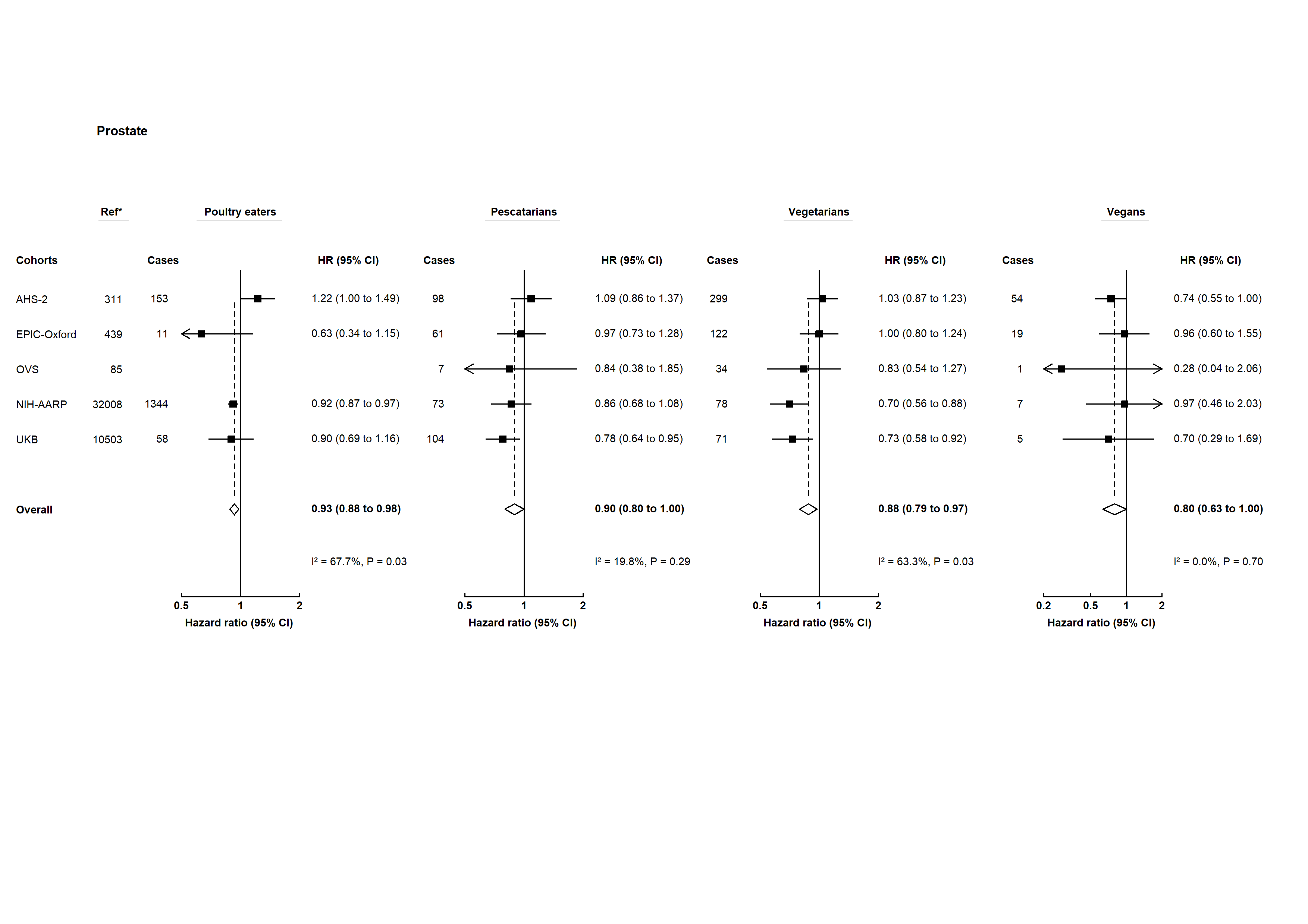


## Supplementary Figure 12. Study-specific and pooled multivariable-adjusted hazard ratios (HRs) and 95% confidence intervals comparing the risk of prostate cancer in poultry eaters, pescatarians and vegetarians to that in meat eaters.

All models used age as the underlying time variable and are stratified by region or method of recruitment and sex, and adjusted for living with partner, educational status, ethnic group, height, body mass index, cigarette smoking, physical activity, alcohol intake, history of diabetes, and history of screening with prostate specific antigen. Confidence intervals extending beyond the axis are marked with arrows.

*Ref, number of cases in the reference group of meat eaters.

Abbreviations: AHS-2, Adventist Health Study-2; EPIC, European Prospective Investigation into Cancer and Nutrition; NIH-AARP, National Institutes of Health-AARP Diet and Health Study; OVS, Oxford Vegetarian Study; UKB, UK Biobank.


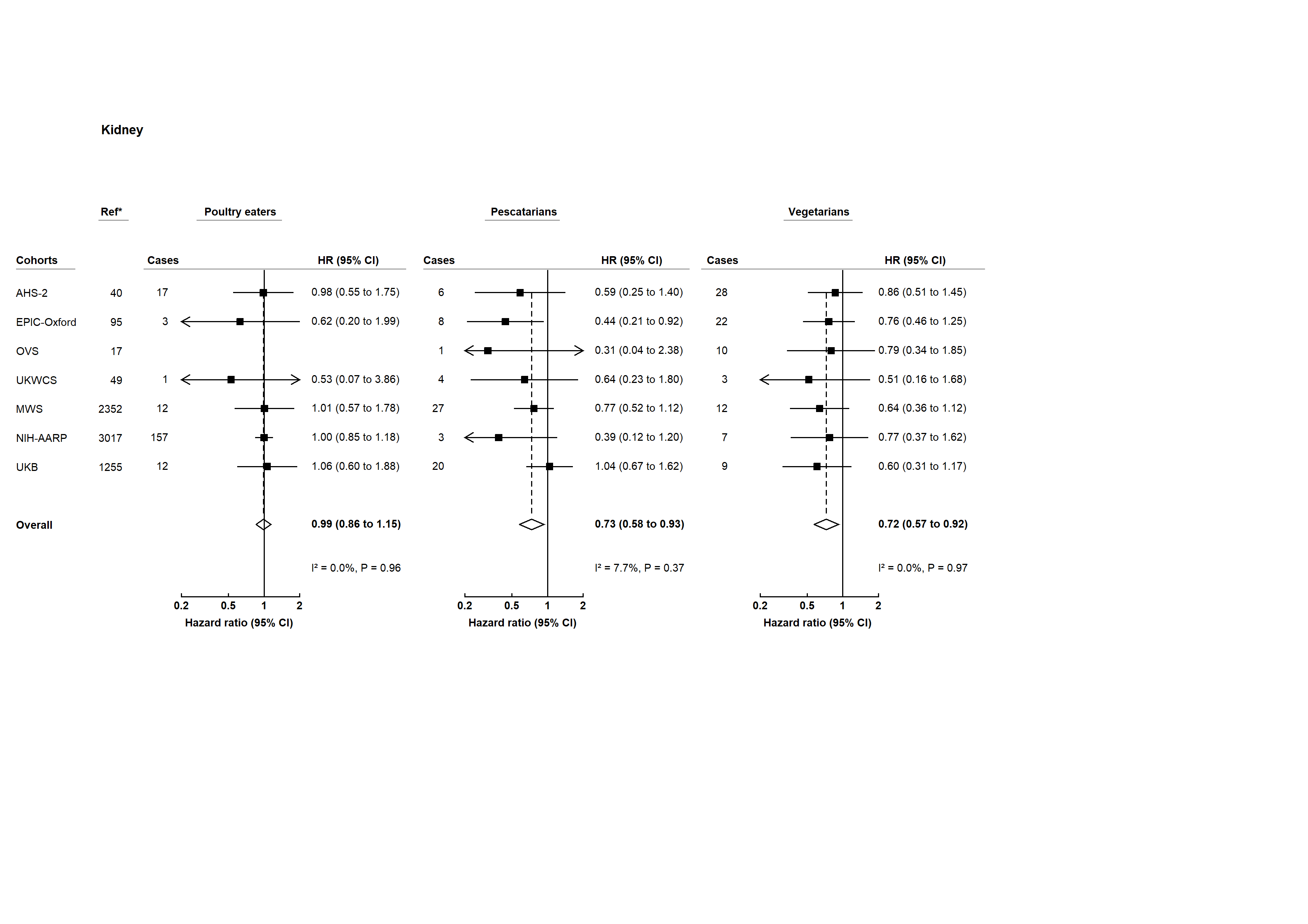


## Supplementary Figure 13. Study-specific and pooled multivariable-adjusted hazard ratios (HRs) and 95% confidence intervals comparing the risk of kidney cancer in poultry eaters, pescatarians and vegetarians to that in meat eaters.

No results are shown for vegans because there were fewer than 10 cases across all cohorts. All models used age as the underlying time variable and are stratified by region or method of recruitment and sex, and adjusted for living with partner, educational status, ethnic group, height, body mass index, cigarette smoking, physical activity, alcohol intake, history of diabetes, parity, and ever use of hormone replacement therapy. Confidence intervals extending beyond the axis are marked with arrows.

*Ref, number of cases in the reference group of meat eaters.

Abbreviations: AHS-2, Adventist Health Study-2; EPIC, European Prospective Investigation into Cancer and Nutrition; MWS, Million Women Study; NIH-AARP, National Institutes of Health-AARP Diet and Health Study; OVS, Oxford Vegetarian Study; UKWCS, UK Women’s Cohort Study; UKB, UK Biobank.


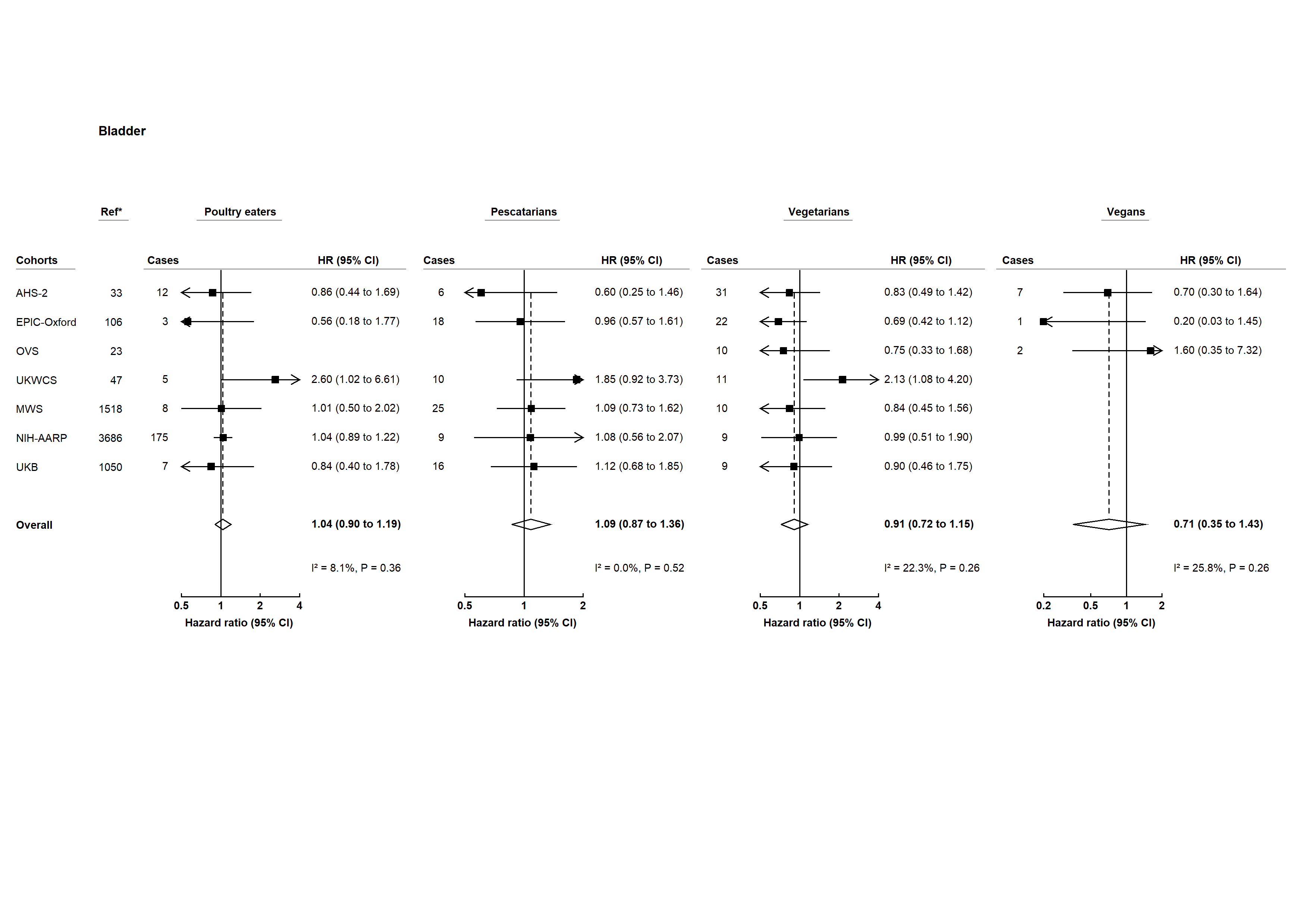


## Supplementary Figure 14. Study-specific and pooled multivariable-adjusted hazard ratios (HRs) and 95% confidence intervals comparing the risk of bladder cancer in poultry eaters, pescatarians and vegetarians to that in meat eaters.

All models used age as the underlying time variable and are stratified by region or method of recruitment and sex, and adjusted for living with partner, educational status, ethnic group, height, body mass index, cigarette smoking, physical activity, alcohol intake, history of diabetes, parity, and ever use of hormone replacement therapy. Confidence intervals extending beyond the axis are marked with arrows.

*Ref, number of cases in the reference group of meat eaters.

Abbreviations: AHS-2, Adventist Health Study-2; EPIC, European Prospective Investigation into Cancer and Nutrition; MWS, Million Women Study; NIH-AARP, National Institutes of Health-AARP Diet and Health Study; OVS, Oxford Vegetarian Study; UKWCS, UK Women’s Cohort Study; UKB, UK Biobank.


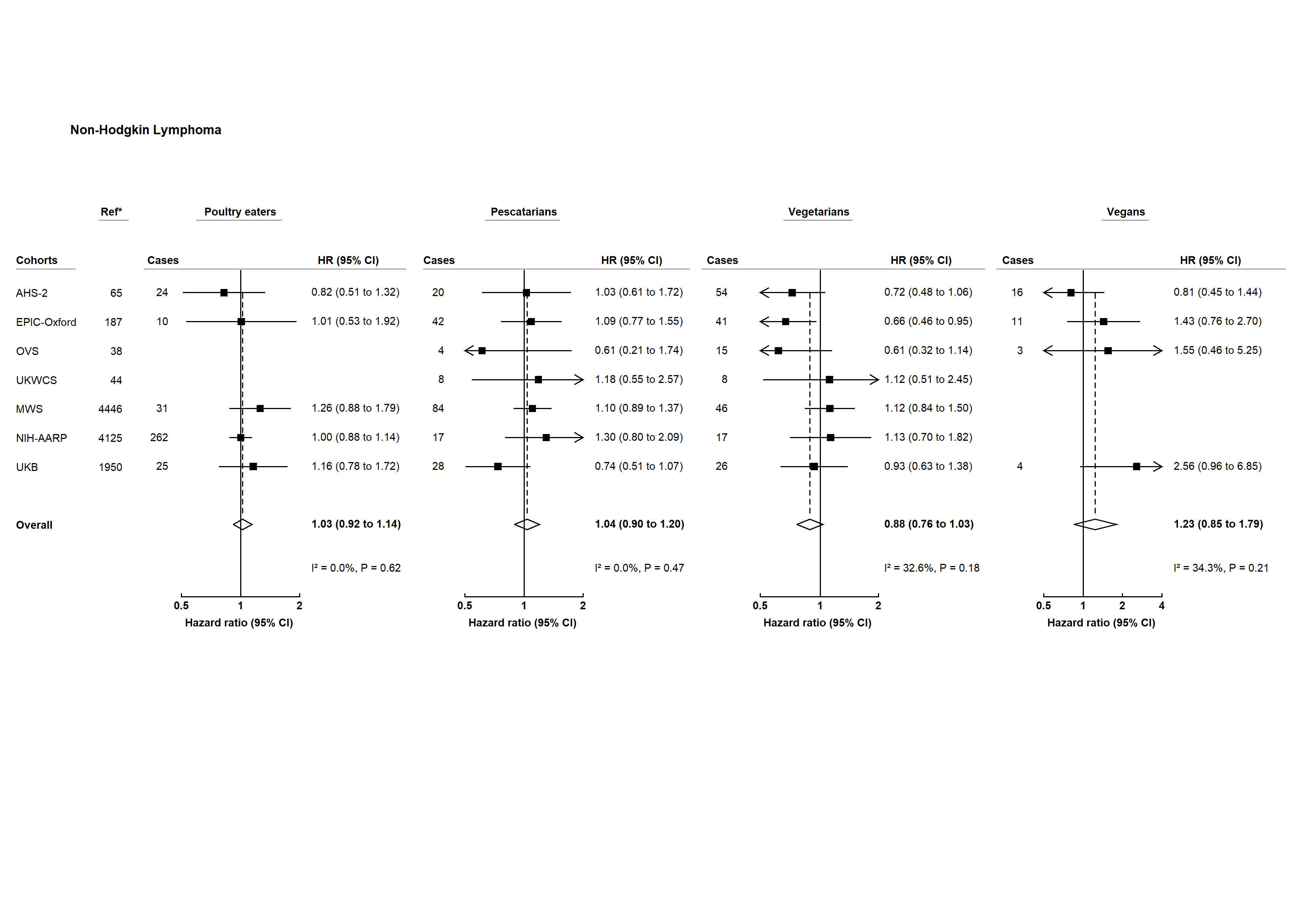


## Supplementary Figure 15. Study-specific and pooled multivariable-adjusted hazard ratios (HRs) and 95% confidence intervals comparing the risk of non-Hodgkin lymphoma in poultry eaters, pescatarians and vegetarians to that in meat eaters.

All models used age as the underlying time variable and are stratified by region or method of recruitment and sex, and adjusted for living with partner, educational status, ethnic group, height, body mass index, cigarette smoking, physical activity, alcohol intake, history of diabetes, parity, and ever use of hormone replacement therapy. Confidence intervals extending beyond the axis are marked with arrows.

*Ref, number of cases in the reference group of meat eaters.

Abbreviations: AHS-2, Adventist Health Study-2; EPIC, European Prospective Investigation into Cancer and Nutrition; MWS, Million Women Study; NIH-AARP, National Institutes of Health-AARP Diet and Health Study; OVS, Oxford Vegetarian Study; UKWCS, UK Women’s Cohort Study; UKB, UK Biobank.


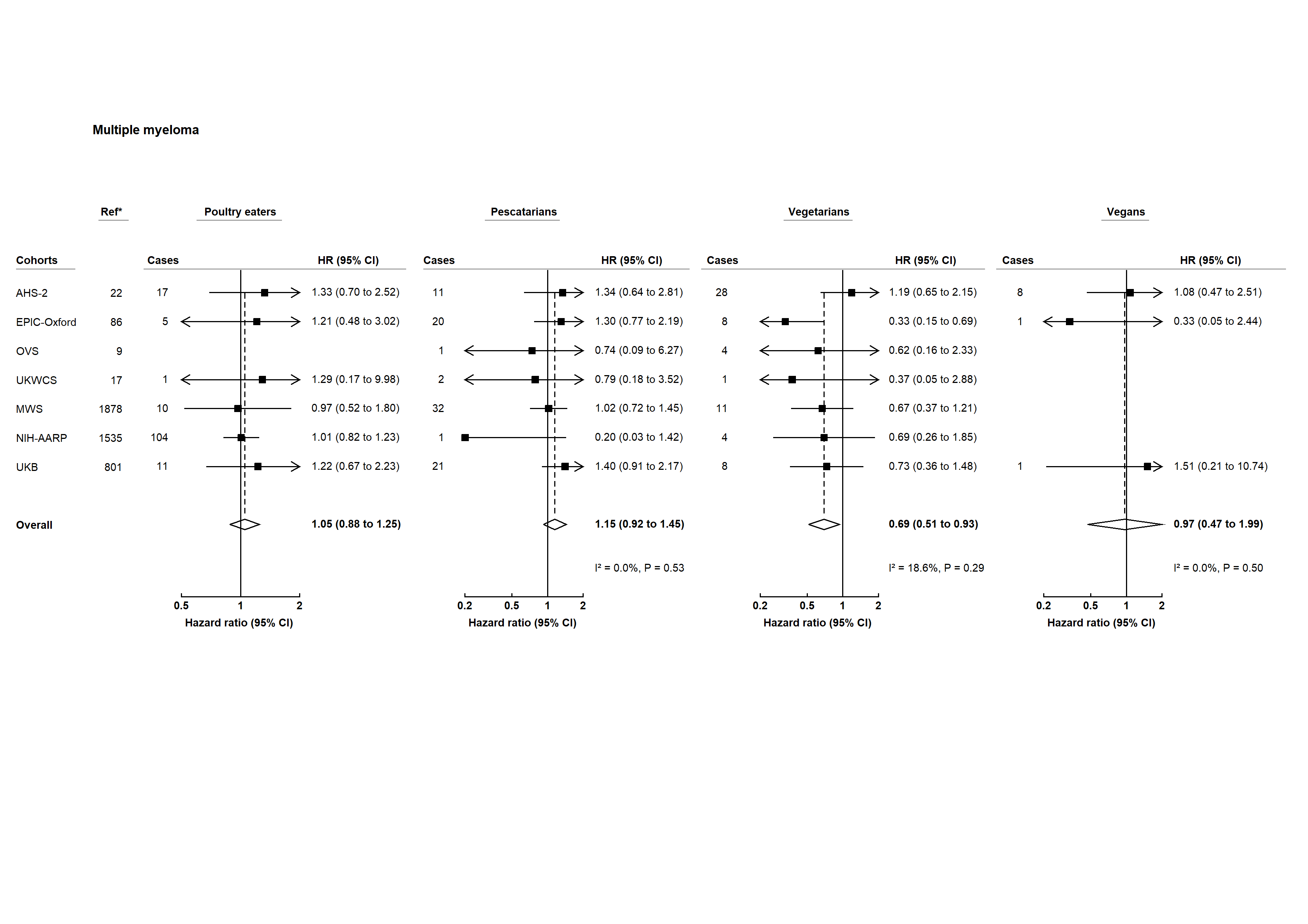


## Supplementary Figure 16. Study-specific and pooled multivariable-adjusted hazard ratios (HRs) and 95% confidence intervals comparing the risk of multiple myeloma in poultry eaters, pescatarians and vegetarians to that in meat eaters.

All models used age as the underlying time variable and are stratified by region or method of recruitment and sex, and adjusted for living with partner, educational status, ethnic group, height, body mass index, cigarette smoking, physical activity, alcohol intake, history of diabetes, parity, and ever use of hormone replacement therapy. Confidence intervals extending beyond the axis are marked with arrows.

*Ref, number of cases in the reference group of meat eaters.

Abbreviations: AHS-2, Adventist Health Study-2; EPIC, European Prospective Investigation into Cancer and Nutrition; MWS, Million Women Study; NIH-AARP, National Institutes of Health-AARP Diet and Health Study; OVS, Oxford Vegetarian Study; UKWCS, UK Women’s Cohort Study; UKB, UK Biobank.

##

##
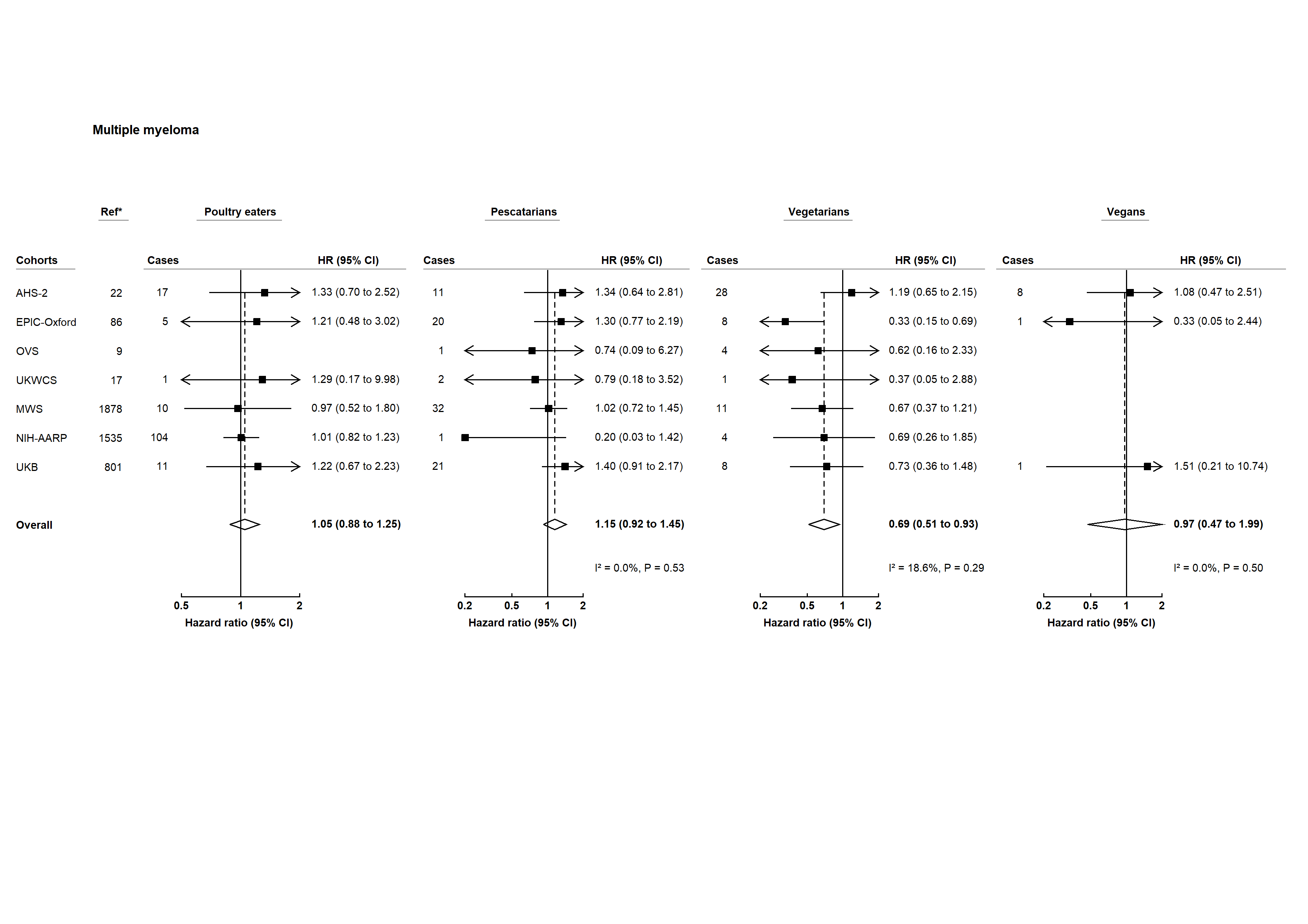

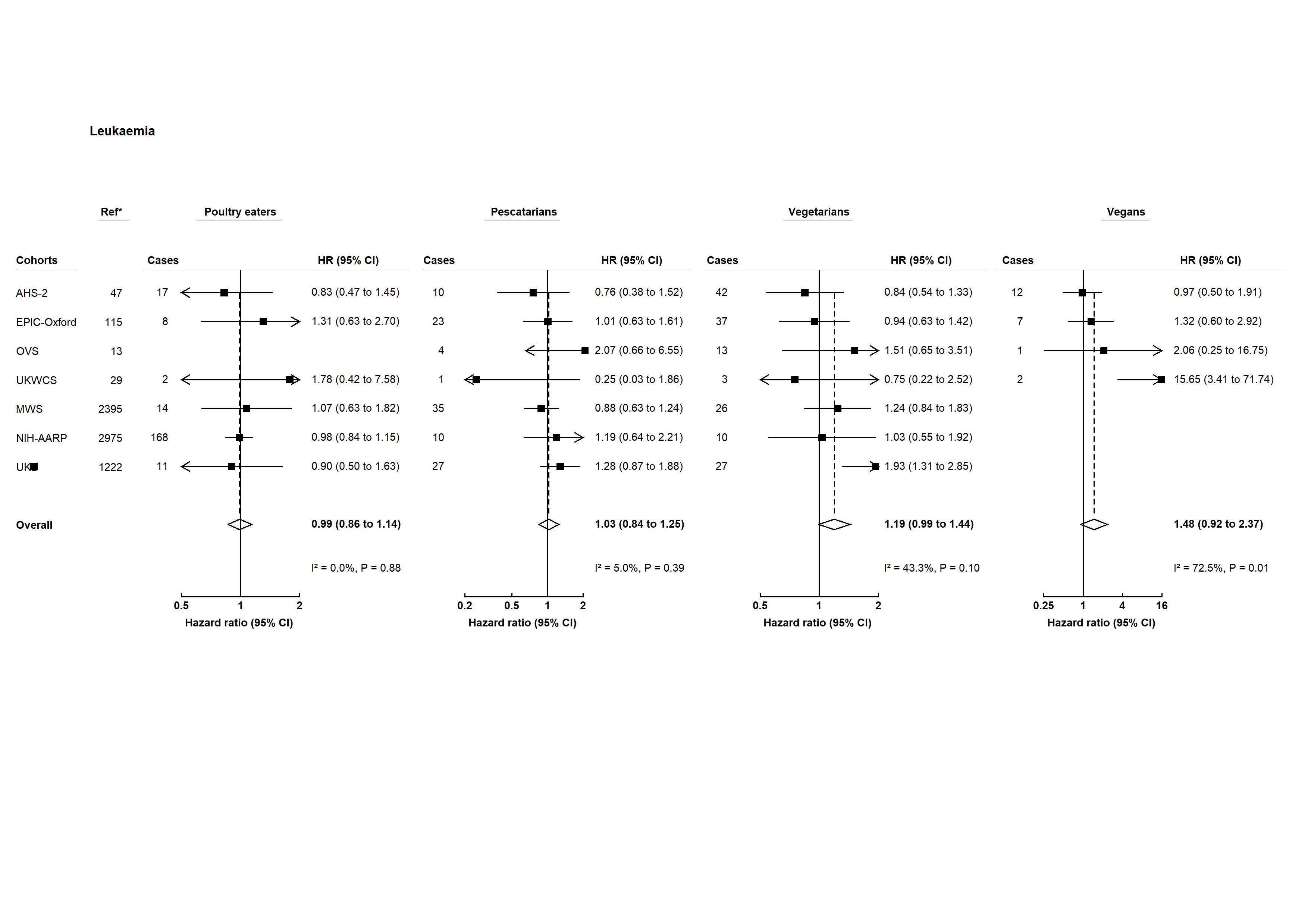
Supplementary Figure 17. Study-specific and pooled multivariable-adjusted hazard ratios (HRs) and 95% confidence intervals comparing the risk of leukaemia in poultry eaters, pescatarians and vegetarians to that in meat eaters.

All models used age as the underlying time variable and are stratified by region or method of recruitment and sex, and adjusted for living with partner, educational status, ethnic group, height, body mass index, cigarette smoking, physical activity, alcohol intake, history of diabetes, parity, and ever use of hormone replacement therapy. Confidence intervals extending beyond the axis are marked with arrows.

*Ref, number of cases in the reference group of meat eaters.

Abbreviations: AHS-2, Adventist Health Study-2; EPIC, European Prospective Investigation into Cancer and Nutrition; MWS, Million Women Study; NIH-AARP, National Institutes of Health-AARP Diet and Health Study; OVS, Oxford Vegetarian Study; UKWCS, UK Women’s Cohort Study; UKB, UK Biobank.
