# Supplementary material for: Vegetarian diets and cancer risk: pooled analysis of 1.8 million women and men in nine prospective studies on three continents
Source: Br J Cancer. 2026 Feb 27;134(8):1218–29. doi: 10.1038/s41416-025-03327-4 (PMC13035945; doi:10.1038/s41416-025-03327-4)
Supplement: Supplementary file 2 — Vegetarian diets and cancer risk: pooled analysis of 1.8 million women and men in nine prospective studies on three continents. Supplementary Table 3 [file 41416_2025_3327_MOESM2_ESM.pdf]

Supplementary Table 3

		MAIN ANALYSIS					WITHOUT BMI ADJUSTMENT			CASES AFTER 4 YEARS ONLY			NEVER SMOKERS ONLY		
**Site**	**DietGroup**	**HR**	**P-val**	**I^2**	**I^2-P-val**	**Cases**	**HR**	**P-val**	**Cases**	**HR**	**P-val**	**Cases**	**HR**	**P-val**	**Cases**
bladder	poultry	1.04 (0.90-1.19)	0.626	8	0.364	210	1.02 (0.88-1.17)	0.832	210	0.95 (0.80-1.13)	0.549	141	1.01 (0.76-1.34)	0.923	54
bladder	pescatarian	1.09 (0.87-1.36)	0.472	0	0.522	84	1.06 (0.85-1.33)	0.617	84	1.20 (0.94-1.54)	0.146	71	1.25 (0.89-1.76)	0.190	38
bladder	vegetarian	0.91 (0.72-1.15)	0.433	22	0.259	102	0.90 (0.71-1.13)	0.350	102	0.97 (0.75-1.27)	0.837	77	1.14 (0.80-1.62)	0.479	58
bladder	vegan	0.71 (0.35-1.43)	0.338	26	0.260	10	0.73 (0.37-1.45)	0.372	10			8			7
breast	poultry	0.96 (0.91-1.01)	0.130	33	0.165	1670	0.93 (0.89-0.98)	0.007	1670	0.95 (0.89-1.01)	0.097	1164	1.00 (0.93-1.08)	0.895	859
breast	pescatarian	0.93 (0.88-0.98)	0.008	0	0.552	1405	0.90 (0.85-0.95)	<0.001	1405	0.98 (0.92-1.04)	0.459	1094	0.94 (0.87-1.01)	0.104	779
breast	vegetarian	0.91 (0.86-0.97)	0.003	0	0.688	1499	0.89 (0.84-0.94)	<0.001	1499	0.96 (0.89-1.02)	0.192	1154	0.95 (0.88-1.02)	0.176	970
breast	vegan	0.86 (0.71-1.04)	0.129	13	0.326	117	0.81 (0.67-0.98)	0.029	117	0.81 (0.64-1.02)	0.070	81	0.80 (0.63-1.03)	0.084	75
colorectal	poultry	0.93 (0.86-1)	0.046	0	0.806	767	0.90 (0.84-0.97)	0.007	767	0.90 (0.82-0.98)	0.015	545	0.92 (0.82-1.03)	0.166	342
colorectal	pescatarian	0.85 (0.77-0.93)	<0.001	17	0.293	445	0.83 (0.75-0.91)	<0.001	445	0.87 (0.78-0.96)	0.009	357	0.89 (0.78-1.01)	0.080	247
colorectal	vegetarian	1.03 (0.94-1.13)	0.582	47	0.067	622	1.01 (0.92-1.1)	0.914	622	1.07 (0.96-1.19)	0.213	486	1.00 (0.89-1.14)	0.939	363
colorectal	vegan	1.40 (1.12-1.75)	0.003	13	0.329	93	1.32 (1.06-1.64)	0.014	93	1.25 (0.95-1.64)	0.115	60	1.67 (1.28-2.18)	<0.001	71
distal_colon	poultry	0.83 (0.70-0.98)	0.025	0	0.893	151	0.79 (0.67-0.94)	0.006	151	0.70 (0.56-0.87)	0.001	89	0.91 (0.71-1.17)	0.466	71
distal_colon	pescatarian	0.80 (0.65-0.98)	0.030	0	0.572	95	0.77 (0.63-0.95)	0.015	95	0.86 (0.68-1.08)	0.201	76	0.94 (0.72-1.23)	0.668	55
distal_colon	vegetarian	0.95 (0.78-1.17)	0.652	60	0.020	123	0.94 (0.76-1.15)	0.529	123	1.05 (0.84-1.32)	0.673	100	0.97 (0.73-1.29)	0.817	71
distal_colon	vegan	1.63 (0.99-2.69)	0.054	15	0.321	19	1.57 (0.96-2.56)	0.073	19	1.44 (0.74-2.81)	0.278	12	3.00 (1.75-5.12)	<0.001	18
endometrial	poultry	1.07 (0.95-1.2)	0.256	0	0.444	320	0.93 (0.83-1.04)	0.209	320	1.05 (0.92-1.21)	0.464	224	0.97 (0.83-1.15)	0.743	166
endometrial	pescatarian	1.01 (0.88-1.16)	0.885	0	0.707	232	0.86 (0.75-0.99)	0.031	232	1.05 (0.90-1.23)	0.504	189	1.05 (0.89-1.25)	0.570	148
endometrial	vegetarian	0.86 (0.74-1.01)	0.060	0	0.853	226	0.75 (0.65-0.88)	<0.001	226	0.88 (0.74-1.04)	0.133	176	0.97 (0.81-1.16)	0.743	179
endometrial	vegan	1.02 (0.66-1.58)	0.924	0	0.902	25	0.74 (0.48-1.14)	0.173	25	1.07 (0.65-1.77)	0.794	19	1.16 (0.70-1.92)	0.573	20
leukaemia	poultry	0.99 (0.86-1.14)	0.892	0	0.877	220	0.96 (0.83-1.1)	0.537	220	1.02 (0.87-1.19)	0.815	173	0.92 (0.74-1.15)	0.471	95
leukaemia	pescatarian	1.03 (0.84-1.25)	0.797	5	0.389	110	1.00 (0.82-1.22)	0.988	110	1.02 (0.82-1.28)	0.834	85	1.08 (0.83-1.41)	0.578	62
leukaemia	vegetarian	1.19 (0.99-1.44)	0.064	43	0.102	158	1.16 (0.97-1.4)	0.113	158	1.40 (1.14-1.72)	0.001	132	1.26 (0.99-1.6)	0.066	104
leukaemia	vegan	1.48 (0.92-2.37)	0.108	72	0.012	22	1.39 (0.88-2.22)	0.161	22	1.96 (1.15-3.33)	0.013	18	1.93 (1.06-3.51)	0.032	15
liver	poultry	1.08 (0.85-1.37)	0.519	0	0.804	80	1.01 (0.80-1.27)	0.959	80	1.12 (0.87-1.45)	0.369	67	1.10 (0.75-1.6)	0.621	32
liver	pescatarian	1.04 (0.75-1.45)	0.802	0	0.884	39	0.98 (0.71-1.36)	0.907	39	1.10 (0.76-1.6)	0.608	30	1.13 (0.70-1.83)	0.606	18
liver	vegetarian	0.84 (0.58-1.21)	0.346	0	0.556	41	0.80 (0.55-1.16)	0.239	41	0.70 (0.43-1.13)	0.140	21	0.89 (0.45-1.75)	0.738	16
liver	vegan					3			3			2			0
lung	poultry	0.83 (0.77-0.89)	0.000	0.00	0.966	830	0.85 (0.79-0.91)	0.000	830	0.83 (0.77-0.9)	0.000	629	0.99 (0.82-1.21)	0.941	113
lung	pescatarian	0.82 (0.72-0.92)	0.001	0.00	0.696	267	0.84 (0.75-0.95)	0.006	267	0.84 (0.73-0.96)	0.011	217	1.00 (0.77-1.31)	1.000	61
lung	vegetarian	0.95 (0.84-1.07)	0.411	9.28	0.358	333	0.98 (0.87-1.11)	0.763	333	1.04 (0.90-1.2)	0.572	251	1.07 (0.83-1.38)	0.594	93
lung	vegan	0.85 (0.58-1.26)	0.425	0.00	0.429	29	0.89 (0.60-1.31)	0.563	29	1.15 (0.74-1.77)	0.542	24	1.00 (0.53-1.9)	0.999	13
mouth_pharynx	poultry	1.06 (0.89-1.27)	0.486	33	0.175	141	1.11 (0.93-1.32)	0.246	141	1.16 (0.95-1.41)	0.146	113	1.20 (0.89-1.6)	0.227	53
mouth_pharynx	pescatarian	1.12 (0.88-1.43)	0.351	49	0.069	74	1.18 (0.92-1.5)	0.192	74	1.24 (0.95-1.63)	0.115	62	1.13 (0.76-1.68)	0.548	29
mouth_pharynx	vegetarian	0.84 (0.64-1.09)	0.182	0	0.450	77	0.86 (0.67-1.12)	0.274	77	0.83 (0.60-1.15)	0.257	52	1.05 (0.72-1.54)	0.797	39
mouth_pharynx	vegan					4			4			3			3
myeloma	poultry	1.05 (0.88-1.25)	0.580	0	0.953	148	1.03 (0.87-1.22)	0.735	148	1.13 (0.93-1.37)	0.204	120	1.11 (0.87-1.42)	0.382	78
myeloma	pescatarian	1.15 (0.92-1.45)	0.209	0	0.531	88	1.13 (0.90-1.41)	0.285	88	1.21 (0.94-1.56)	0.134	70	1.14 (0.84-1.54)	0.408	49
myeloma	vegetarian	0.69 (0.51-0.93)	0.016	19	0.288	64	0.69 (0.51-0.93)	0.014	64	0.75 (0.54-1.05)	0.093	48	0.76 (0.53-1.1)	0.149	46
myeloma	vegan	0.97 (0.47-1.99)	0.931	0	0.505	10	1.03 (0.50-2.09)	0.941	10			7			8
non-hodg_lymph	poultry	1.03 (0.92-1.14)	0.655	0	0.622	352	1.01 (0.90-1.12)	0.896	352	1.04 (0.92-1.18)	0.547	269	1.06 (0.90-1.24)	0.497	174
non-hodg_lymph	pescatarian	1.04 (0.90-1.2)	0.633	0	0.475	203	1.02 (0.88-1.18)	0.778	203	1.05 (0.89-1.23)	0.599	158	1.08 (0.89-1.3)	0.438	120
non-hodg_lymph	vegetarian	0.88 (0.76-1.03)	0.125	33	0.179	207	0.88 (0.75-1.02)	0.098	207	0.97 (0.82-1.16)	0.740	162	0.86 (0.70-1.05)	0.145	138
non-hodg_lymph	vegan	1.23 (0.85-1.79)	0.267	34	0.207	34	1.24 (0.86-1.79)	0.258	34	1.39 (0.91-2.13)	0.126	27	1.03 (0.63-1.66)	0.919	21
oesophagus_ac	poultry	0.83 (0.58-1.18)	0.293	0	0.882	32	0.74 (0.52-1.06)	0.102	32	0.84 (0.56-1.27)	0.409	24	1.25 (0.68-2.29)	0.468	11
oesophagus_ac	pescatarian	0.55 (0.29-1.03)	0.062	0	0.405	10	0.49 (0.26-0.91)	0.024	10			9			4
oesophagus_ac	vegetarian	0.59 (0.32-1.11)	0.100	0	0.762	12	0.54 (0.29-1.01)	0.052	12	0.66 (0.34-1.29)	0.225	11			4

oesophagus_ac	vegan					2			2			2			0
oesophagus_scc	poultry	1.36 (0.92-2.02)	0.127	32	0.219	26	1.52 (1.02-2.25)	0.038	26	1.30 (0.82-2.07)	0.192	19	1.92 (1.00-3.69)	0.050	10
oesophagus_scc	pescatarian	1.17 (0.78-1.77)	0.444	6	0.346	24	1.31 (0.87-1.98)	0.193	24	1.33 (0.85-2.07)	0.211	21			8
oesophagus_scc	vegetarian	1.93 (1.30-2.87)	0.001	0	0.890	31	2.15 (1.44-3.2)	<0.001	31	2.09 (1.34-3.26)	0.001	25	2.93 (1.82-4.73)	<0.001	22
oesophagus_scc	vegan					1			1			0			0
ovarian	poultry	1.00 (0.87-1.16)	0.996	12	0.336	213	0.99 (0.85-1.14)	0.860	213	1.02 (0.86-1.2)	0.861	155	1.15 (0.96-1.39)	0.138	129
ovarian	pescatarian	0.94 (0.81-1.1)	0.447	0	0.596	184	0.93 (0.80-1.08)	0.314	184	1.05 (0.88-1.23)	0.603	153	0.85 (0.69-1.06)	0.148	92
ovarian	vegetarian	0.97 (0.82-1.14)	0.713	0	0.667	209	0.95 (0.81-1.11)	0.509	209	0.99 (0.82-1.19)	0.920	163	1.03 (0.85-1.26)	0.737	143
ovarian	vegan	0.94 (0.52-1.68)	0.832	48	0.145	13	0.88 (0.49-1.57)	0.665	13	0.89 (0.46-1.74)	0.738	10	0.94 (0.49-1.81)	0.859	11
pancreatic	poultry	0.92 (0.80-1.05)	0.225	23	0.258	227	0.89 (0.78-1.03)	0.108	227	0.89 (0.76-1.04)	0.139	181	0.82 (0.66-1.02)	0.076	92
pancreatic	pescatarian	0.99 (0.83-1.19)	0.955	0	0.866	127	0.96 (0.80-1.15)	0.649	127	1.03 (0.84-1.25)	0.799	107	1.03 (0.80-1.33)	0.830	67
pancreatic	vegetarian	0.79 (0.65-0.97)	0.024	0	0.539	126	0.76 (0.62-0.93)	0.009	126	0.77 (0.61-0.97)	0.024	95	0.84 (0.64-1.1)	0.198	79
pancreatic	vegan	1.06 (0.64-1.76)	0.829	60	0.042	17	0.98 (0.59-1.61)	0.928	17	1.05 (0.55-2.02)	0.881	10	0.85 (0.44-1.63)	0.619	11
prostate	poultry	0.93 (0.88-0.98)	0.007	68	0.026	1566	0.94 (0.89-0.99)	0.015	1566	0.91 (0.86-0.97)	0.003	1087	0.98 (0.91-1.06)	0.598	723
prostate	pescatarian	0.90 (0.80-1)	0.055	20	0.289	343	0.90 (0.80-1.01)	0.065	343	0.90 (0.79-1.03)	0.135	238	0.93 (0.80-1.08)	0.340	197
prostate	vegetarian	0.88 (0.79-0.97)	0.011	63	0.028	604	0.88 (0.80-0.97)	0.012	604	0.93 (0.82-1.04)	0.210	427	0.99 (0.87-1.13)	0.922	440
prostate	vegan	0.80 (0.63-1)	0.053	0	0.700	86	0.79 (0.63-0.99)	0.039	86	0.93 (0.82-1.05)	0.257	55	0.95 (0.72-1.26)	0.741	64
proximal_colon	poultry	1.05 (0.95-1.16)	0.369	27	0.235	401	1.02 (0.92-1.13)	0.680	401	1.07 (0.95-1.2)	0.288	312	0.98 (0.84-1.15)	0.803	179
proximal_colon	pescatarian	0.80 (0.68-0.94)	0.005	0	0.744	167	0.78 (0.67-0.91)	0.002	167	0.84 (0.71-1)	0.045	143	0.76 (0.61-0.94)	0.012	90
proximal_colon	vegetarian	1.08 (0.93-1.25)	0.338	46	0.083	247	1.04 (0.90-1.21)	0.606	247	1.12 (0.95-1.32)	0.185	196	1.01 (0.83-1.23)	0.932	154
proximal_colon	vegan	1.35 (0.94-1.94)	0.107	0	0.789	36	1.20 (0.84-1.72)	0.313	36	1.22 (0.76-1.94)	0.415	20	1.56 (1.03-2.37)	0.034	30
rectal	poultry	0.88 (0.76-1.02)	0.098	46	0.098	180	0.87 (0.75-1.01)	0.076	180	0.85 (0.70-1.02)	0.075	120	0.98 (0.77-1.23)	0.834	82
rectal	pescatarian	0.97 (0.83-1.15)	0.760	36	0.152	155	0.97 (0.82-1.14)	0.705	155	0.98 (0.81-1.18)	0.842	120	1.06 (0.85-1.32)	0.621	84
rectal	vegetarian	1.07 (0.91-1.27)	0.406	0	0.483	195	1.07 (0.91-1.26)	0.425	195	1.13 (0.94-1.36)	0.198	159	1.14 (0.91-1.42)	0.254	116
rectal	vegan	1.78 (1.23-2.57)	0.002	0	0.733	35	1.76 (1.22-2.53)	0.002	35	1.77 (1.16-2.72)	0.009	25	1.94 (1.18-3.18)	0.008	21
renal	poultry	0.99 (0.86-1.15)	0.906	0	0.957	202	0.92 (0.80-1.06)	0.255	202	0.87 (0.74-1.04)	0.128	139	1.09 (0.88-1.35)	0.433	96
renal	pescatarian	0.73 (0.58-0.93)	0.012	8	0.369	69	0.67 (0.53-0.85)	0.001	69	0.70 (0.53-0.92)	0.011	53	0.72 (0.51-1.02)	0.066	34
renal	vegetarian	0.72 (0.57-0.92)	0.009	0	0.969	91	0.67 (0.53-0.85)	0.001	91	0.70 (0.54-0.93)	0.012	66	0.64 (0.46-0.88)	0.007	50
renal	vegan					6			6			5			4
stomach	poultry	0.90 (0.73-1.12)	0.361	0	0.573	91	0.88 (0.71-1.09)	0.248	91	0.82 (0.62-1.06)	0.133	59	0.82 (0.58-1.17)	0.275	38
stomach	pescatarian	0.92 (0.69-1.24)	0.592	0	0.690	48	0.90 (0.67-1.2)	0.456	48	1.01 (0.73-1.42)	0.933	37	1.20 (0.82-1.75)	0.343	30
stomach	vegetarian	0.83 (0.63-1.1)	0.201	0	0.622	67	0.80 (0.61-1.07)	0.130	67	0.99 (0.72-1.38)	0.970	52	0.98 (0.68-1.42)	0.932	43
stomach	vegan					6			6			1			5
total_colon	poultry	0.96 (0.88-1.04)	0.304	37	0.162	587	0.93 (0.85-1.01)	0.080	587	0.93 (0.84-1.03)	0.148	425	0.93 (0.81-1.06)	0.249	260
total_colon	pescatarian	0.80 (0.71-0.9)	<0.001	1	0.419	290	0.78 (0.69-0.88)	<0.001	290	0.83 (0.73-0.95)	0.006	237	0.83 (0.71-0.97)	0.023	163
total_colon	vegetarian	0.99 (0.88-1.11)	0.833	0	0.498	409	0.96 (0.86-1.07)	0.461	409	1.04 (0.92-1.18)	0.506	327	0.95 (0.82-1.11)	0.551	247
total_colon	vegan	1.26 (0.95-1.68)	0.108	0	0.430	58	1.15 (0.87-1.52)	0.322	58	1.06 (0.74-1.52)	0.734	35	1.65 (1.20-2.27)	0.002	50
